# EBV renders B cells susceptible to HIV-1 in humanized mice

**DOI:** 10.26508/lsa.202000640

**Published:** 2020-06-23

**Authors:** Donal McHugh, Renier Myburgh, Nicole Caduff, Michael Spohn, Yik Lim Kok, Christian W Keller, Anita Murer, Bithi Chatterjee, Julia Rühl, Christine Engelmann, Obinna Chijioke, Isaak Quast, Mohaned Shilaih, Victoria P Strouvelle, Kathrin Neumann, Thomas Menter, Stephan Dirnhofer, Janice KP Lam, Kwai F Hui, Simon Bredl, Erika Schlaepfer, Silvia Sorce, Andrea Zbinden, Riccarda Capaul, Jan D Lünemann, Adriano Aguzzi, Alan KS Chiang, Werner Kempf, Alexandra Trkola, Karin J Metzner, Markus G Manz, Adam Grundhoff, Roberto F Speck, Christian Münz

**Affiliations:** 1 Viral Immunobiology, Institute of Experimental Immunology, University of Zürich, Zürich, Switzerland; 2 Department of Medical Oncology and Hematology, University and University Hospital of Zürich, Zürich, Switzerland; 3 Heinrich Pette Institute, Leibniz Institute for Experimental Virology, Hamburg, Germany; 4 Division of Infectious Diseases and Hospital Epidemiology, University Hospital of Zürich, Zürich, Switzerland; 5 Neuroinflammation, Institute of Experimental Immunology, University of Zürich, Zürich, Switzerland; 6 Cellular Immunotherapy, Institute of Experimental Immunology, University of Zürich, Zürich, Switzerland; 7 Institute of Pathology and Medical Genetics, University Hospital of Basel, Basel, Switzerland; 8 Institute of Medical Virology, University of Zürich, Zürich, Switzerland; 9 Department of Paediatrics and Adolescent Medicine, Li Ka Shing Faculty of Medicine, Queen Mary Hospital, The University of Hong Kong, Pokfulam, Hong Kong; 10 Institute of Neuropathology, University Hospital of Zurich, Zurich, Switzerland; 11 Kempf und Pfaltz Histologische Diagnostik AG, Zürich, Switzerland

## Abstract

HIV and EBV are human pathogens that cause a considerable burden to worldwide health. In combination, these viruses are linked to AIDS-associated lymphomas. We found that EBV, which transforms B cells, renders them susceptible to HIV-1 infection in a CXCR4 and CD4-dependent manner in vitro and that CXCR4-tropic HIV-1 integrates into the genome of these B cells with the same molecular profile as in autologous CD4^+^ T cells. In addition, we established a humanized mouse model to investigate the in vivo interactions of EBV and HIV-1 upon coinfection. The respective mice that reconstitute human immune system components upon transplantation with CD34^+^ human hematopoietic progenitor cells could recapitulate aspects of EBV and HIV immunobiology observed in dual-infected patients. Upon coinfection of humanized mice, EBV/HIV dual-infected B cells could be detected, but were susceptible to CD8^+^ T-cell–mediated immune control.

## Abstract (precis)

A proportion of EBV^+^ B cells are infectible by CXCR4-tropic HIV-1 in vitro and in vivo, and these HIV-infected B cells can transmit infection to previously virus-naïve humanized mice.

## Introduction

EBV-associated lymphomas are a considerable threat for individuals infected with the HIV type-1 (HIV-1) and constitute an AIDS–defining condition (1). With more than 90% of the adult human population being latently infected with the oncogenic γ-herpesvirus EBV (2), coinfection with EBV is the norm rather than an exception for people infected with HIV-1 (3). Nevertheless, studies exploring experimental HIV-1–EBV coinfections are lacking.

After primary infection, EBV establishes a life-long latent infection within the memory B-cell compartment with minimal viral gene expression. To gain access to this persistence reservoir, the virus relies on the expression of up to eight latency-associated genes (EBV nuclear antigens [EBNA] -1, -2, -3A-C, and -LP, and latent membrane proteins [LMP] -1 and -2) and two clusters of small noncoding RNAs (EBV-encoded RNAs [EBERs] and viral miRNAs), which promote proliferation, transformation, and confer resistance to apoptosis and immune eradication. Despite the strong B cell–transforming capacity of the virus, only few infected individuals develop EBV-associated lymphomas because of continuous restriction by EBV-specific immune responses. This immune control seems to be mainly mediated by T cells because iatrogenic T-cell–directed immune suppression leads to the increased occurrence of EBV-associated B cell lymphoproliferative diseases, which can be cured by adoptive transfer of in vitro–expanded EBV-specific T cells (4).

Chronic infection with HIV-1 results in the progressive failure of the immune system that culminates in AIDS (5). The rate of EBV association with non-Hodgkin lymphomas (NHL) in patients with AIDS is higher than with NHL in immunocompetent individuals, reaching up to 90% and 100% for systemic immunoblastic diffuse large B-cell lymphoma and central nervous system lymphoma, respectively (1, 4). The emergence of EBV-associated central nervous system lymphomas correlates with the HIV-1–induced loss of EBV-specific CD4^+^ T cells rather than overall CD4^+^ T-cell loss (6). It has been suggested that this loss of EBV-specific CD4^+^ T cells leads to an exhausted CD8^+^ T-cell population that can no longer control EBV-mediated lymphoproliferation, resulting in a progression toward NHLs (7).

Although current combined anti-retroviral therapy (cART) leads to sustained suppression of HIV RNA copy numbers in patients, poor adherence results in viral rebound because of the existence of latently HIV-1–infected cells (8). The main targets for HIV-1 replication are CD4^+^ T cells, and these represent the major cell subset harbouring silent HIV-1 (8). However, HIV-1 infection is not solely restricted to CD4^+^ T cells, and other cell types are also susceptible to the virus (8, 9, 10). Susceptibility to HIV-1 infection requires the co-expression of CD4 and either CCR5 (R5) or CXCR4 (X4) receptors on the cellular surface (11). In vitro, HIV-1 can infect IL-4– and/or CD40L-activated primary B cells because of the expression of CD4 and CXCR4, and infection may be enhanced in PMA-activated B cells via binding of HIV-1 in immune complexes by complement receptors CD21 and CD35 (12, 13, 14, 15). Also, HIV-1 infection of EBV-transformed and EBV-negative B cell lines has been described previously (16, 17, 18, 19, 20, 21). Although direct interactions between HIV-1 and B cells have been reported several decades ago (22, 23), there are, to our knowledge, no reports of HIV-1 replication in B cells in vivo or experimental coinfection of HIV-1 with EBV in vivo, as reviewed in reference 24.

In this study, we aimed to characterize the influence of EBV infection on HIV-1 susceptibility and possible HIV-1 integration in EBV-transformed B cells for lentiviral reservoir generation, as well as its influence on viral and host gene transcription in comparison to CD4^+^ T cells in vitro. Humanized mice have been successfully used to separately investigate HIV-1 and EBV infection, pathogenesis, and immune control, and we and others could recapitulate the protective value of CD4^+^ or CD8^+^ T cells against EBV-mediated lymphoproliferation in these models (25, 26, 27, 28). Here, we modelled and investigated the effect of HIV-1 on EBV-specific immune control via coinfection of humanized mice. This in vivo model then allowed us to examine whether EBV-transformed primary B cells serve as target cells for HIV-1 replication upon dual virus infection.

## Results

### EBV-transformed B cells are permissive to X4-tropic but not R5-tropic HIV-1 infection in vitro

**Figure 1.**
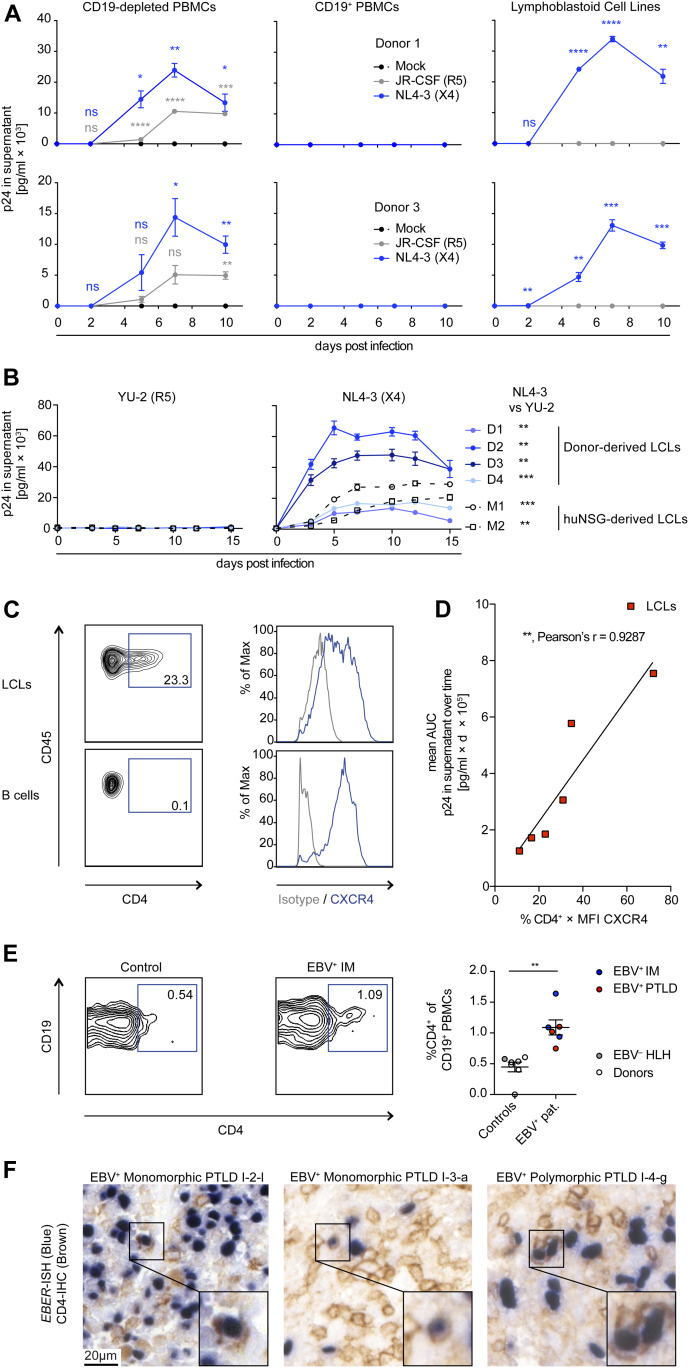
X4-tropic HIV-1 replicates in EBV-transformed B cells in vitro. **(A)** Quantification of p24 via ELISA in supernatants collected over 10 d from in vitro HIV-1–infected CD19^+^ B-cell–depleted PBMCs, purified CD19^+^ B cells and lymphoblastoid cell lines (LCLs) from two donors. Cells were either mock-infected, infected with JR-CSF (R5-tropic HIV-1), or NL4-3 (X4-tropic HIV-1). Data from two donors are depicted. Adjusted *P*-value summaries for comparison of NL4-3 and JR-CSF versus Mock are indicated in blue and grey, respectively, from two-tailed unpaired *t* tests, corrected by the Holm–Sidak method. **(B)** Quantification of p24 in supernatants collected over 15 d from HIV-1–infected LCLs derived from four donors and two EBV-infected humanized mice reconstituted with fetal liver-derived CD34^+^ cells. Cells were infected with YU-2 (R5-tropic HIV-1) or NL4-3. The area under the curve (AUC, p24 in the supernatant versus time postinfection) was compared via two-tailed unpaired *t* test with Welch’s correction. **(C)** Representative flow cytometry plots and histograms of LCLs and autologous purified B cells stained for CD45, CD4, and CXCR4. **(D)** Correlation of NL4-3 HIV-1 replication in different LCLs with the level of CXCR4 and CD4 surface expression before infection. HIV-1 replication was approximated via analyzing the AUC for each LCL as shown in (B). Relative surface expression of HIV entry receptors for each LCL was approximated by multiplying the frequency of CD4^+^ cells with the median fluorescence intensity of CXCR4 as determined by flow cytometry immune phenotyping. Correlation, **P = 0.0074, Pearson’s r = 0.9287. **(E)** Representative flow cytometry immune phenotyping logarithmic contour plots and quantification of CD4 surface expression on CD19^+^ B cells from controls (healthy blood donors and 1 EBV^−^ hemophagocytic lymphohistiocytosis [HLH] patient) and EBV^+^ infectious mononucleosis or EBV^+^ posttransplant lymphoproliferative disorder (PTLDs) patients. Events were pre-gated on single cells/lymphocytes/CD3^−^/CD19^+^ cells. **P = 0.001 (Mann–Whitney test). **(F)** Dual *EBER* in situ hybridization (blue) CD4 immunohistochemistry (IHC, brown) on tissue microarrays of two cases of EBV^+^ PTLD. I-2-l and I-3-a are separate sections from the same PTLD. Scale bar: 20 μm. Inserts are a 2× magnification of a section of the main image. In (A, B, C), data are represented as mean ± SEM and were performed in triplicate. Results representative of four donors. *P*-values are reported as ns > 0.05, * < 0.05, ** < 0.01, *** < 0.001, **** < 0.0001.

**Figure S1.**
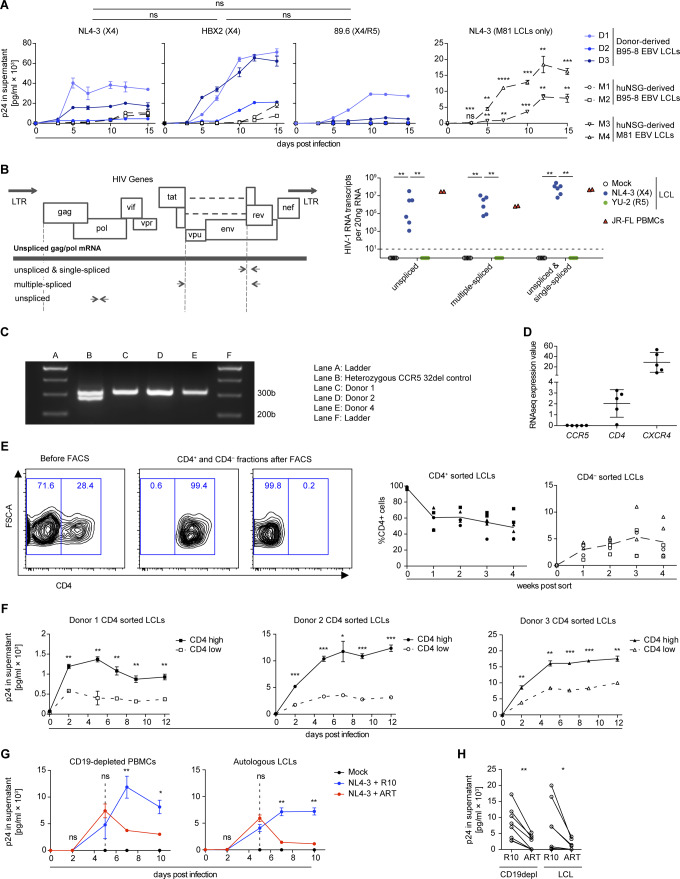
HIV-1 infects lymphoblastoid cell lines (LCLs) in a X4- and CD4-dependent manner. **(A)** Quantification of p24 by ELISA in supernatants collected from in vitro NL4-3, HBX2, and 89.6 HIV-1–infected LCLs. The mean AUC (p24 in the supernatant versus time postinfection) from five LCLs was compared via the Mann–Whitney test. M81 EBV LCLs derived from EBV-infected humanized mice generated from two individual human fetal liver donors. Adjusted *P*-value summaries for comparison of NL4-3–infected M81 LCLs versus Mock are depicted from two-tailed unpaired *t* tests, corrected by the Holm–Sidak method. Data are represented as mean ± SEM and were performed in triplicates. **(B)** Wild-type HIV-1 gene map showing position of primers used to detect unspliced, single-spliced, and multiple-spliced HIV-1 mRNA transcripts (left). Quantification of HIV-1 RNA transcripts per 20 ng of RNA in six LCLs (four derived from donors and two from EBV-infected humanized mice) and one PBMC donor was performed 15 d postinfection (right). LCLs were infected with X4-tropic NL4-3 and R5-tropic YU-2 HIV-1 strains, and control PBMCs were infected with JR-FL R5-tropic HIV-1 strain (two-tailed Fischer’s exact test for presence versus absence of HIV-1–specific transcripts). **(C)** Representative PCR results for genotyping CCR5 alleles in LCLs donors used in this study. Amplification of the homozygous wild-type allele (CCR5^+/+^) results in a single band of 311 bp. Amplification of the heterozygous allele (CCR5^+/delta32^) results in two bands of 311 and 279 bp. **(D)** Mean expression values as determined by RNA-seq for *CCR5*, *CD4*, and *CXCR4* transcripts in five different LCLs (two humanized mice-derived and three donor-derived LCLs) are plotted. Each plotted value is the mean expression value (=RPKM) from three biological replicates (RNA sequencing data from McHugh et al (53)). **(E)** Sorted CD4^+^ and CD4^−^ LCLs populations and subsequent quantification of the frequency of CD4 surface expression over 4 wk of in vitro culture. Two independent experiments with three donors; means are indicated with connecting lines. **(F)** Quantification of p24 by ELISA in supernatants collected from in vitro NL4-3 HIV-1–infected CD4 high and CD4 low LCLs from three donors. The infection was performed in triplicate 4 wk after sorting for CD4. Data are represented as mean ± SEM. Adjusted *P*-value summaries for comparison of CD4 high versus low LCLs are depicted from two-tailed unpaired *t* tests, corrected by the Holm–Sidak method. **(G)** Anti-retroviral treatment (ART) of in vitro NL4-3 HIV-1–infected LCLs and autologous CD19^+^ B-cell–depleted PBMCs. 5 d postinfection, the cells were treated with AZT and Efaverenz or medium (R10). Data are represented as mean ± SEM and were performed in triplicate. Results are representative of four donors. Adjusted *P*-value summaries for comparison of ART-treated versus R10 treated are depicted from two-tailed unpaired *t* tests, corrected by the Holm–Sidak method. **(H)** Summary of mean p24 concentrations in culture supernatants of HIV-1–infected LCLs and autologous CD19-depleted PBMCs cultured in R10 with or without ART treatment. CD19depl **P = 0.001; LCL *P = 0.049 (two-tailed paired *t* test). **(A, B, F, H)** *P < 0.05, **P < 0.01, ***P < 0.001, ****P < 0.0001.

We analyzed the capacity of HIV-1 to infect EBV-transformed B cells (lymphoblastoid cell lines; LCLs) in comparison to autologous PBMCs. As expected, we observed viral replication over time, quantified by the HIV-1 p24 antigen in the supernatant, in CD19-depleted PBMCs but not CD19-purified B cells inoculated with the R5-tropic (JR-CFS) or X4-tropic (NL4-3) HIV-strains (Fig 1A). Autologous LCLs generated by in vitro EBV (B95-8 strain) infection of B cells were able to sustain X4-tropic HIV-1 (NL4-3 and HBX2) and dual-tropic HIV-1 (89.6) replication, as were LCLs derived from EBV-infected (B95.8 or M81 strain) humanized mice (Figs 1A and B and S1A and B). These results are in line with data reported previously for several EBV-positive and EBV-negative B cell lines (14, 22, 29, 30). Despite the fact that none of the LCL donors was a carrier of the CCR5delta32 deletion (Fig S1C), LCLs were not susceptible to R5-tropic HIV-1 strains (YU-2 and JR-CSF), evidenced by the lack of p24 in the culture supernatant and the absence of HIV-1 mRNA transcript splice variants (Figs 1B and S1B). In line with the selective susceptibility of LCLs to X4-tropic HIV-1 infection, we found that a large fraction of the LCLs expressed CD4 on their surface and retained expression of CXCR4 at lower levels upon EBV transformation, whereas transcript levels of CCR5 were very low in selectively sequenced LCLs as previously reported (Figs 1C and S1D) (31, 32, 33). The combined surface expression of CD4 and CXCR4 of individual LCLs correlated strongly with HIV-1 replication over time in vitro (Fig 1D). Furthermore, upon FACS, pure populations of CD4^+^ and CD4^−^ LCLs either lost or gained CD4 expression, respectively, within days of in vitro culture, indicating that the CD4^+^ cells are unlikely derived from a clonal population. The resultant CD4^high^ LCL subcultures were more susceptible to X4-tropic HIV-1 replication compared with the autologous CD4^low^ subcultures (Fig S1E and F). In addition, X4-tropic HIV-1 replication was efficiently suppressed by anti-retroviral treatment (ART: Efavirenz & AZT) in vitro in both donor LCLs and autologous CD4^+^ T cells (Fig S1G and H). To assess if EBV infection can induce CD4 expression in humans, we investigated PBMCs from healthy blood donors, patients with infectious mononucleosis, that is, the primary symptomatic infection with EBV, or EBV^+^ B cell posttransplant lymphoproliferative disorders (PTLDs) and found that patients with EBV-associated diseases had more frequent CD4^+^ B cells than controls (Fig 1E and Table S1). Moreover, in a separate cohort of 10 EBV^+^ B cell PTLDs, we found evidence of CD4 surface expression on EBV-infected (EBER^+^) cells in two patients (Fig 1F and Table S2). These previously described PTLDs were also positive for CXCR4 (34). In summary, EBV transformation can induce B-cell susceptibility to HIV-1 infection in vitro in a CD4- and CXCR4-dependent manner, similar to IL-4 and/or CD40L stimulation of B cells as shown previously (12, 14) and CD4 can be detected on peripheral blood B cells and lymphoma cells in EBV-associated diseases.

Table S1 PBMCs stained for CD4 expression on B cells.

Table S2 Posttransplant DLBCL patient tissue microarrays co-positive for CD4 expression and *EBER1 RNA*.

### X4-tropic HIV-1 host genome integration profile in LCLs is similar to autologous T cells

**Figure 2.**
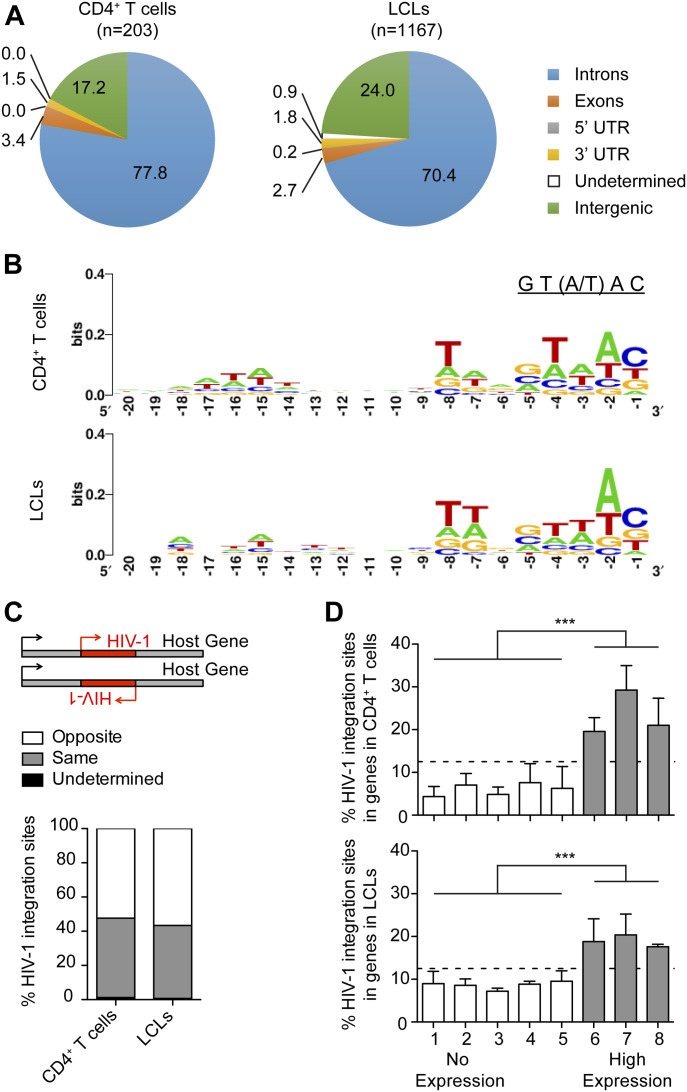
X4-tropic HIV-1 host genome integration profile in lymphoblastoid cell lines (LCLs) and autologous T cells. **(A)** Distribution of HIV-1 integration sites in the host genome comparing CD4^+^ T cells and autologous LCLs 2 d post in vitro infection with NL4-3 (n = number of integration sites). **(B)** Nucleotide sequence consensus upstream of HIV-1 5′LTR in the host genome. **(C)** Transcription orientation of intragenic HIV-1 relative to the host gene. HIV-1 integration events in loci of transcript variants or more than one gene with different features of interest are classified as undetermined. **(D)** Frequency of HIV-1 integrations in genes of autologous donor CD4^+^ T cells and LCLs. Host genes in noninfected LCLs and CD4^+^ T cells were ranked from non-expressed to highly expressed genes based on reads per kilobase of RNA transcript per million mapped reads (RPKM). The host genes were grouped into eight bins of equal size based on RPKM. Only the grey bins contain genes with an RPKM value greater than 0. The relative distribution of the total HIV-1 integration sites among the eight bins is summarized for the CD4^+^ T cells and LCLs. ***P < 0.0001 (Chi-squared test). **(A, B, C, D)** Data derived from three donors: two LCLs and three CD4^+^ T cells each with two separate HIV-1 or mock infections; two of the T cells were autologous to the investigated LCLs.

HIV-1 proviral integration into the host cell genome is an essential part of the retroviral life cycle and lentiviral reservoir generation (35). Therefore, we investigated HIV-1 integration into the host genome of LCLs and whether integration profiles differ between LCLs and CD4^+^ T cells. Purified CD4^+^ T cells and autologous LCLs enriched for CD4 expression by FACS were infected with X4-tropic HIV-1 for 2 d. HIV-1 integration sites were amplified with a nonrestrictive linear amplification-mediated PCR (nrLAM-PCR) (36, 37), sequenced, and reads were mapped using the Integration Site Analysis Pipeline (37). We found that HIV-1 integrates into the genome of LCLs with an overrepresentation of sites within transcriptional units similar to CD4^+^ T cells (37, 38, 39) (Fig 2A and Table S3) and monocytes/macrophages (37, 40). We observed the HIV-1 signature weakly conserved palindromic nucleotide sequence (GT(A/T)AC) in the host genome upstream of HIV-1 integration in both the CD4^+^ T cells and the LCLs (Fig 2B), as previously described for T cells (37, 41, 42, 43). In line with previous findings (39, 42), we did not observe a preference for the orientation of the HIV-1 provirus relative to the transcriptional direction of host genes in CD4^+^ T cells and LCLs (Fig 2C). To correlate HIV-1 proviral integration with gene transcription in LCLs and CD4^+^ T cells, RNA sequencing was performed with LCLs and autologous CD4^+^ T cells 2 d after infection with X4-tropic HIV-1 or mock infection. Upon ranking host gene expression in mock-infected CD4^+^ T cells and autologous LCLs based on reads per kilobase of RNA transcript per million mapped reads (RPKM), we grouped these genes into eight bins of equal sizes and observed that integration sites were overrepresented in bins containing actively transcribed genes (Fig 2D). These data indicate that HIV-1 favors integration in actively transcribed genes in both LCLs and CD4^+^ T cells confirming previous reports for CD4^+^ T-cell infection (38, 44). We also observed a trend toward reduced integration in the most transcriptionally active set of genes. Because of their more central location in the nucleus, highly transcribed genes may be less accessible for viral integration, which often occurs close to the nuclear pore (44). HIV-1 integration in LCLs was found to be enriched in genes for endosomal trafficking (gene ontology [GO] term: 0006895, Golgi to endosome transport, 9.89-fold enriched, false discovery rate [FDR] = 0.003; GO term: 0032456, endocytic recycling, 7.32-fold enriched, FDR = 0.011), whereas HIV-1 integration in CD4^+^ T cells was primarily enriched for genes of the choline transport (GO term: 0015871, 51.24-fold enriched, FDR = 0.019; Table S2). However, in both LCLs and T cells, HIV-1 proviruses also targeted genes that regulate mitotic cell cycle phase transition (GO term: 1901990; LCLs: 2.15-fold enriched, FDR = 0.013, T cells: 3.69, FDR = 0.048), indicating frequent integration into cell cycle–associated genes. These genes are actively transcribed in LCLs as well as activated T cells and are thus likely more accessible to HIV-1 integration (Table S4). It remains, however, unclear if these integration events alter the expression of the respective host genes and thereby affect lymphocyte growth behaviour. No HIV-1 integrations into the EBV genome were detected. In summary, the integration profiles of X4-tropic HIV-1 in the host genomes of CD4^+^ T cells and LCLs follow the same pattern.

Table S3 HIV-1 integration sites in lymphoblastoid cell line and CD4^+^ T-cell genome.

Table S4 GO term analysis of genes with HIV-1 integration.

### CD8^+^ T cells expand but do not control EBV during EBV/HIV dual infection of humanized mice

**Figure 3.**
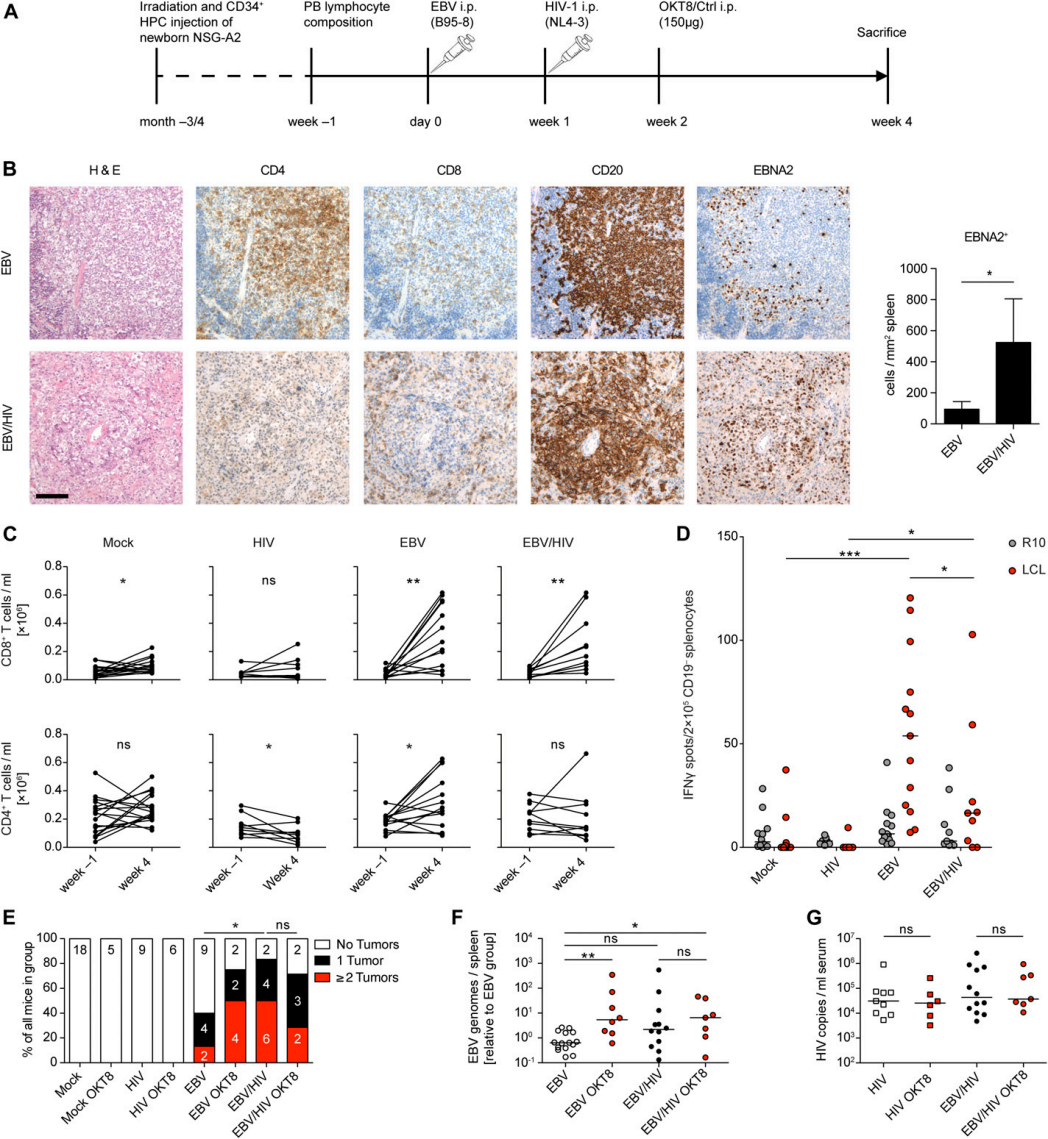
EBV/HIV dual-infection of humanized mice. **(A)** Experimental setup: newborn NSG-A2 mice were irradiated and received intrahepatic injection of human CD34^+^ hematopoietic progenitor cells. Before infection, baseline blood lymphocyte composition was determined by flow cytometry and mice were grouped. At day 0, mice were infected i.p. with EBV or PBS and 1 wk later with HIV-1 or PBS. At week 2 post-infection, mice received a single injection of a CD8-depleting monoclonal antibody (OKT8) or an isotype control antibody. In one experiment, mice were left untreated. Mice were euthanized 4 wk post-EBV infection. **(B)** Representative histological panel of formalin-fixed, paraffin-embedded spleen sections from EBV and EBV/HIV–infected huNSG-A2 mice depicting H&E, CD4, CD8, CD20 and EBNA2 IHC stainings. Scale bar: 100 μm. Quantification of EBNA2^+^ cells per square millimeter with n = 7 and 5 mice, respectively. *P = 0.030 (Mann–Whitney test, MWT). **(C)** Number of CD8^+^ and CD4^+^ T cells per ml blood at week −1 and at week 4. Mock; CD8^+^ *P = 0.049. HIV; CD4^+^ *P = 0.042. EBV; CD8^+^ **P = 0.001 and CD4^+^ *P = 0.021. EBV/HIV; CD8^+^ **P = 0.007 (two-tailed paired *t* test). **(D)** EBV-specific T cell ELISpot assay. Mean IFNγ release was quantified upon co-culture of CD19-depleted splenocytes derived from humanized mice of the indicated groups with autologous EBV-transformed B cells (lymphoblastoid cell lines or LCLs) or medium (R10). Mock versus EBV ***P < 0.001; HIV versus EBV/HIV *P = 0.013; EBV versus EBV/HIV *P = 0.045 (MWT). **(E)** Presence of macroscopically visible EBV-associated tumors in animals from the indicated experimental groups. *P = 0.016 (MWT for tumor score). **(F)** Total splenic EBV DNA burden was determined for each mouse by qPCR for EBV BamHI W fragment and plotted relative to EBV-infected animals. EBV versus EBV OKT8 treated *P = 0.005; EBV versus EBV/HIV *P* = 0.060; EBV versus EBV/HIV OKT8 treated **P = 0.035; EBV/HIV versus EBV/HIV OKT8 treated *P* = 0.583 (MWT). **(G)** Serum HIV RNA copy numbers were determined by RT-qPCR at week 4 (MWT). **(B, C, D, E, F, G)** Represents pooled data from three experiments. **(F, G)** Individual values for each mouse and median are depicted.

**Figure S2.**
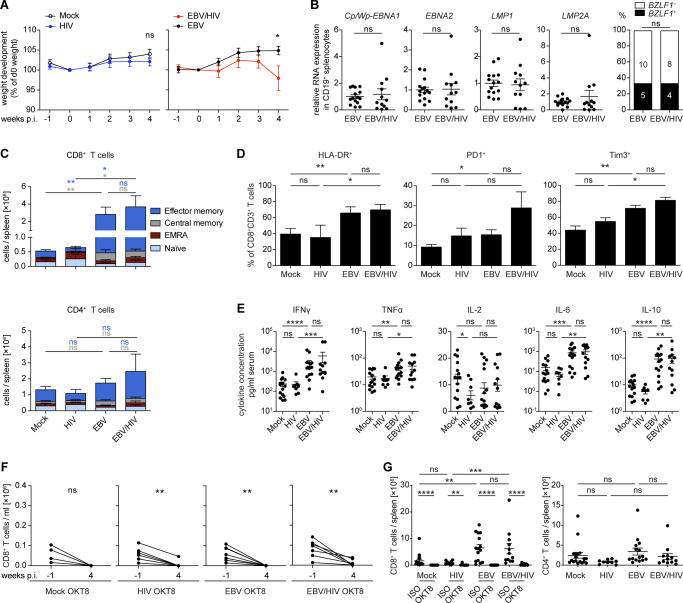
Characteristics of humanized mice during EBV/HIV dual-infection. **(A)** Weight development comparing Mock versus HIV *P* = 0.326 and EBV versus EBV/HIV *P = 0.029—infected mice relative to day 0 and up to week 4 post EBV infection (two-tailed unpaired *t* test at week 4). **(B)** Quantification of EBV transcripts *Cp/Wp-EBNA1 P* = 0.608, *EBNA2 P* = 0.643, *LMP1 P* = 0.714, and *LMP2A P* = 0.696 via RT-qPCR in CD19^+^ splenocytes of EBV-infected and EBV/HIV–infected mice. Data normalized to the mean of the EBV group values (Mann–Whitney test [MWT]). Frequency of detection of the *BZLF1* transcript in CD19^+^ B cells from individual mice (Fischer’s exact test). **(C)** Differentiation status of splenic CD8^+^ and CD4^+^ T cells at termination. Mean ± SEM total number of CD8^+^ and CD4^+^ T cells per spleen is shown. Effector Memory: CD62L^−^ CD45RA^−^. Central Memory: CD62L^+^ CD45RA^−^. EMRA: CD62L^−^ CD45RA^+^. Naive: CD62L^+^ CD45RA^+^ (MWT for Central and Effector Memory). **(D)** Frequency of HLA-DR, PD1, and Tim3 surface expression on splenic CD8^+^ T cells at 4 wk post EBV infection (MWT). **(E)** Quantification of cytokines in the serum of mice 4 wk post EBV infection (MWT). **(F)** Number of CD8^+^ T cells per ml blood for individual mice at week −1 and week 4 in mice that received OKT8 treatment at week 2. Mock OKT8: *P* = 0.064. HIV OKT8: **P = 0.003. EBV OKT8: **P = 0.002. EBV/HIV OKT8: **P = 0.006 (two-tailed paired *t* test). **(G)** Absolute numbers of CD8^+^ and CD4^+^ T cells per spleen in mice from different experimental groups were quantified at week 4 (MWT). **(A, B, C, D, E, F, G)** Data pooled from two to four experiments and in (A, B, C, D, E, G) represented as mean ± SEM, ns *P* > 0.05, *P < 0.05, **P < 0.01, ***P < 0.001, ****P < 0.0001.

Because in vitro infection of cells cannot fully recapitulate the effects of EBV plus HIV-1 dual infection with respect to the induced immune responses, we investigated EBV–HIV-1 interactions in an in vivo model of infection and immune control. NOD-*scid* γ_c_^−/−^ Tg(HLA-A2) (NSG-A2) mice with human immune system components (humanized mice) reconstituted from CD34^+^ hematopoietic progenitor cells (HPCs) were infected with EBV (B95-8 (45)) and 1 wk later with HIV-1 (NL4-3). The experiment was terminated at 4 wk post-EBV infection because of the considerable weight loss of EBV/HIV dual-infected mice (Figs 3A and S2A). Conventional H&E and immunohistochemistry (IHC) staining of splenic sections from dual-infected animals revealed the presence of tumor-like lesions containing CD20^+^ B cells with a high frequency of EBNA2-positive cells and similar CD8^+^ T-cell infiltration compared with EBV-infected mice (Fig 3B). No significant differences were detected in the relative expression of latent and lytic EBV transcripts in non-tumorous splenic B cells isolated from either EBV or EBV/HIV dual-infected mice (Fig S2B). We observed a small but significant loss of CD4^+^ T cells in the blood of HIV-infected mice, an expansion of both CD8^+^ and CD4^+^ T cells in EBV-infected mice, and a similar expansion of CD8^+^ but not CD4^+^ T cells in EBV/HIV dual-infected mice (Fig 3C). Particularly, the memory CD8^+^ T cells were increased, as determined in the spleen (Fig S2C). Further analysis of splenic CD8^+^ T cells revealed a trend towards an activated, but exhausted phenotype in EBV/HIV dual-infected mice with enhanced surface expression of HLA-DR, PD1, and Tim3 (Fig S2D). Although serum cytokine levels of IFNγ, TNFα, IL-6, and IL-10 were similarly high in EBV and EBV/HIV–infected mice (Fig S2E), the ex vivo EBV-specific IFNγ release from CD19-depleted splenocytes challenged with autologous LCLs was lower in dual-infected animals (Fig 3D). These findings mirror data comparing IFNγ release of EBV-specific CD8^+^ T cells derived from HIV-1–infected individuals that either remained long-term asymptomatic or progressed to AIDS due to opportunistic infections (46). As such, despite a comparable CD8^+^ T-cell expansion in EBV and EBV/HIV dual-infected mice, T cells from a dual-infected environment did not seem to react to EBV-infected cells as efficiently. Furthermore, macroscopically visible tumors in the spleen, pancreas, and liver were more frequent in EBV/HIV dual-infected mice than EBV single-infected mice and coincided with a trend towards higher EBV burden in the spleen (Fig 3E and F). Moreover, CD8^+^ T-cell depletion via OKT8 treatment (Fig S2F and G) only exacerbated the EBV disease burden in EBV single-infected mice, whereas in EBV/HIV dual-infected animals, OKT8 treatment did not result in a further increase in tumor burden or EBV viral load (Fig 3E and F). In contrast to a previous study in which HIV-1–specific immune control was compromised upon antibody-mediated CD8^+^ T-cell depletion at 2 and 5–7 wk post-HIV-1 infection (47), in our hands, CD8 depletion 1 wk post-HIV-1 infection did not lead to measurable differences in serum HIV-1 copy numbers at 2 wk post-depletion (Fig 3G). In summary, these data indicate that CD8^+^ T cells expand and can control EBV during EBV-single infection and despite expanding in EBV/HIV dual-infected mice, they seem to partially lose EBV-specific immune control leading to enhanced EBV-disease progression.

### B cells from EBV/HIV dual-infected humanized mice can transmit HIV-1 infection to autologous humanized mice

**Figure 4.**
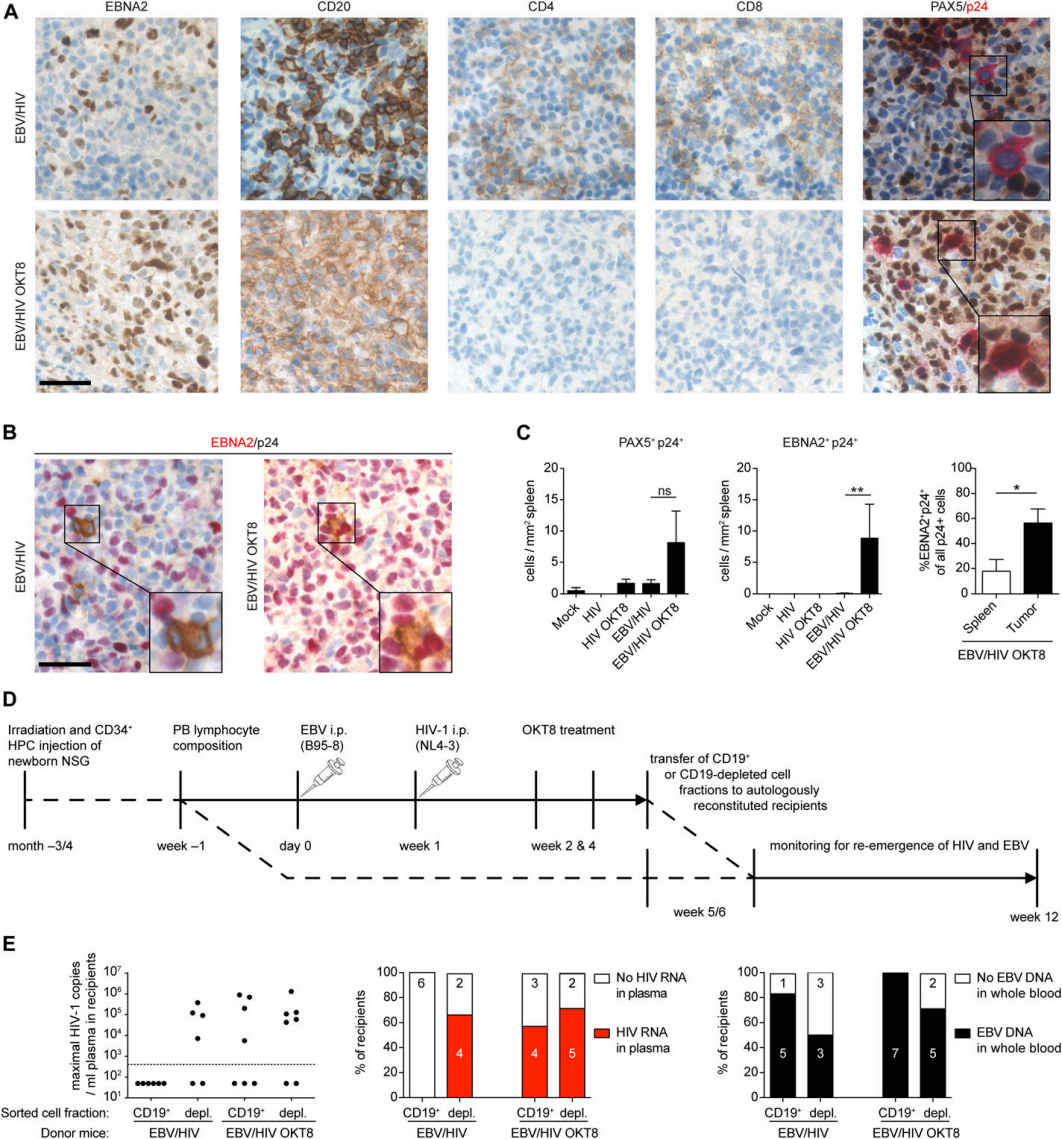
EBV/HIV dual-infected humanized mice replicate HIV-1 in B cells. **(A)** Representative IHC stainings of EBNA2, CD20, CD4, CD8, and co-IHC for PAX5 (brown, nuclear stain) and p24 (red, cytoplasmic stain) of formalin-fixed, paraffin-embedded spleen sections from EBV/HIV–infected and EBV/HIV–infected OKT8-treated mice (EBV/HIV OKT8). **(B)** Representative co-immunohistochemistry stainings for EBNA2 (red, nuclear staining) and p24 (brown, cytoplasmic staining). **(A, B)** Scale bar: 40 μm. Inserts are a 2× magnification of a section of the main image. **(C)** Quantification of PAX5^+^/p24^+^ (left) and EBNA2^+^/p24^+^ (mid) cells in the spleen from the indicated experimental groups and EBNA2^+^/p24^+^ as % of total p24^+^ (right) cells in non-tumorous spleen and tumor tissue within the EBV/HIV OKT8 group. N = respectively; Mock: 4 & 5, HIV: 6 & 3, HIV OKT8: 7 & 5, EBV/HIV: 14 & 12, EBV/HIV OKT8: 15 & 14, “Spleen”: 5, “Tumor”: 4. PAX5^+^/p24^+^ *P* = 0.112; EBNA2^+^/p24^+^: **P = 0.006, Spleen versus Tumor: *P = 0.032 (Mann–Whitney test). **(D)** Experimental setup for cell fraction transfer assay: Newborn NSG mice were irradiated and transplanted with fetal liver derived CD34^+^ hematopoietic progenitor cells. Donor mice were infected with EBV and 1 wk later with X4-tropic HIV-1. At week 2 and 4, donor mice either received the CD8-depleting antibody (OKT8) treatment or PBS. At week 5 or 6, the donors were sacrificed, and splenic lymphocytes were MACS separated into CD19^+^ and CD19-depleted fractions. Recipient mice received either CD19^+^ or CD19-depleted cells i.p. from either EBV/HIV or EBV/HIV OKT8-treated mice. Recipient mice were monitored up to week 12 for HIV-1 RNA in plasma and for EBV DNA in whole blood. **(E)** The highest number of HIV-1 RNA copies measured in plasma of individual-recipient mice and the frequency of recipients with detectable HIV RNA in the plasma and EBV DNA in the peripheral blood after receiving either CD19^+^ or CD19-depleted cells from either EBV/HIV or EBV/HIV OKT8-treated donor mice.

**Figure S3.**
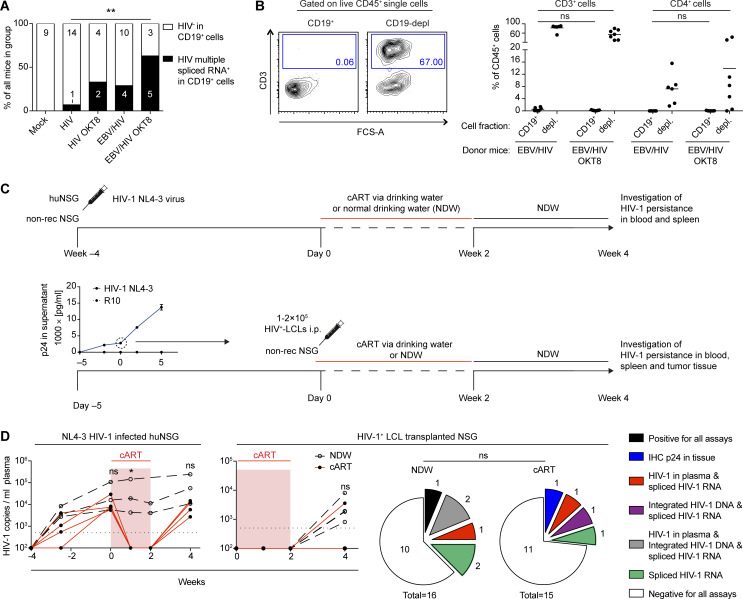
Characteristics of CD19 MACS-purified cell fractions and EBV-transformed B cells as a HIV-1–putative reservoir during combined anti-retroviral therapy (cART) treatment in vivo. **(A)** Frequency of detection of HIV-1 multiple spliced transcripts via RT-qPCR in CD19^+^ B cells from individual-mice from the indicated experimental groups. HIV versus EBV/HIV OKT8 **P = 0.009. Data pooled from four experiments (two-tailed Fisher’s exact test of raw numbers). **(B)** Representative flow cytometry plots (left) indicating T-cell content in CD19^+^ and CD19-depleted (depl.) MACS purified splenocyte fractions and quantification (right) from all mice used as donors for experiments presented in Fig 4 (Mann–Whitney test). **(C)** Scheme of in vivo HIV-1 latency model. Top (controls): At week −4 humanized mice (huNSG) and non-reconstituted NSG mice were infected with NL4-3 live virus preparations. cART (Abacavir, Lamivudine, Dolutegravir) was administered to a subset of huNSG via the drinking water MediDrop Sucralose solution starting on day 0 for 2 wk. The remaining huNSG mice received normal drinking water (NDW) for 2 wk, after which, all mice received NDW for another 2 wk. Bottom: Quantification of p24 by ELISA in supernatants collected from the ex vivo NL4-3 HIV-1–infected lymphoblastoid cell lines (LCLs) (confirmed as B cells via RNA-seq) used for transfer into mice at day 0. cART (Abacavir, Lamivudine, Dolutegravir) was administered to half the mice via the drinking water in Medidrop-sucralose solution starting on day 0 for 2 wk, after which all mice received NDW for another 2 wk. **(D)** Left: Number of HIV-1 RNA copies/ml in the plasma of cART treated and control (NDW) huNSG mice and cART treated and control HIV-1^+^ LCL transplanted NSG mice (Mann–Whitney test). Right: Summary of HIV-1 detection data from cART-treated and control NSG mice transplanted with HIV-1^+^ LCLs, including HIV-1 RNA copies in plasma, p24 immunohistochemistry (IHC) of tissue, integrated HIV-1 DNA (HIV-1 Alu-PCR integration assay), and spliced HIV-1 RNA (HIV-1 specific spliced transcripts via RT-qPCR). Data from two individual experiments (Fischer’s exact test for positive versus negative outcome in any assay).

Based on the ability of HIV-1 to infect LCLs in vitro (Fig 1), we aimed to determine whether HIV-1 infects EBV-infected B cells in humanized mice. Co-IHC detecting the B-cell lineage specific transcription factor PAX5 and the HIV-1 p24 antigen on splenic sections revealed the presence of PAX5^+^ cells positive for cytoplasmic p24 in EBNA2^+^ regions of the spleens of dual-infected animals when CD8^+^ T cells were depleted (Fig 4A). EBNA2^+^/p24^+^ cells observed in dual-infected animals were present at a significantly higher frequency in CD8-depleted mice and comprised a larger fraction of all the HIV-infected cells within tumors than non-tumorous spleen tissue (Fig 4B and C). In line with these findings, multiple spliced HIV-1 RNA transcripts were detected more frequently in purified B-cell fractions from dual-infected CD8-depleted animals (Fig S3A). We aimed to determine whether in vivo EBV/HIV coinfected B cells actively replicate HIV-1 upon transfer to a HIV-1 naïve host. To this end, humanized mice were infected with EBV and 1 wk later with X4-tropic HIV-1, followed by treatment with either CD8^+^ T-cell–depleting antibody or PBS at week 2 and 4 post-EBV infection (Fig 4D). From these dual-infected mice, splenic CD19^+^ B-cell fractions were purified by repeating magnetically activated cell sorting twice, to ensure low T-cell contamination (Fig S3B). These B-cell fractions or an equal number of CD19-depleted splenocytes obtained from dual-infected non–CD8-depleted or dual-infected CD8-depleted donor mice were transferred to individual humanized littermates reconstituted with the same human fetal liver (HFL)-derived CD34^+^ HPCs. Emergence of EBV and HIV was monitored in the blood and plasma of recipient animals for 6–7 wk and, upon euthanizing, in the spleen via IHC for EBNA2 and p24 (Fig 4D). As expected, most of the mice that received the CD4^+^ T-cell containing CD19-depleted fractions from both donor groups developed HIV-1 plasma viral loads (Fig 4E). Supporting our finding that B cells can harbour HIV in EBV/HIV dual-infected humanized mice, some recipients of CD19^+^ B-cell fractions from dual-infected CD8-depleted mice developed detectable HIV-1 RNA loads in the plasma, whereas recipients of B cells from dual-infected non–CD8-depleted mice did not (Fig 4E).

Next, we investigated the potential of EBV^+^ B cells to function as a reservoir for latent HIV-1 infection upon cART treatment in vivo in the absence of T-cell immune control. We infected LCLs derived from humanized mice with HIV-1 and transferred these cells into NSG mice, which we then either treated with cART via the drinking water or normal drinking water for 2 wk, followed by another 2 wk of normal drinking water. To control for effective HIV-1 suppression, humanized mice infected with HIV-1 for 4 wk were subjected to the same 2-wk cART treatment (Fig S3C). We found evidence of HIV-1 persistence in cART-treated mice either by IHC for p24 in the tumor tissue, HIV-1 DNA integration, spliced HIV-1 RNA, or quantification of HIV-1 genomes in the plasma (Fig S3D). In summary, these data indicate that during conditions of reduced CD8^+^ T-cell–mediated immune surveillance, HIV can readily infect EBV-infected B cells, and these cells can either contribute to viremia or potentially serve as a reservoir for HIV-1 during cART treatment.

### HIV-1–infected LCLs are more susceptible to elimination by autologous T-cell clones in vitro

**Figure 5.**
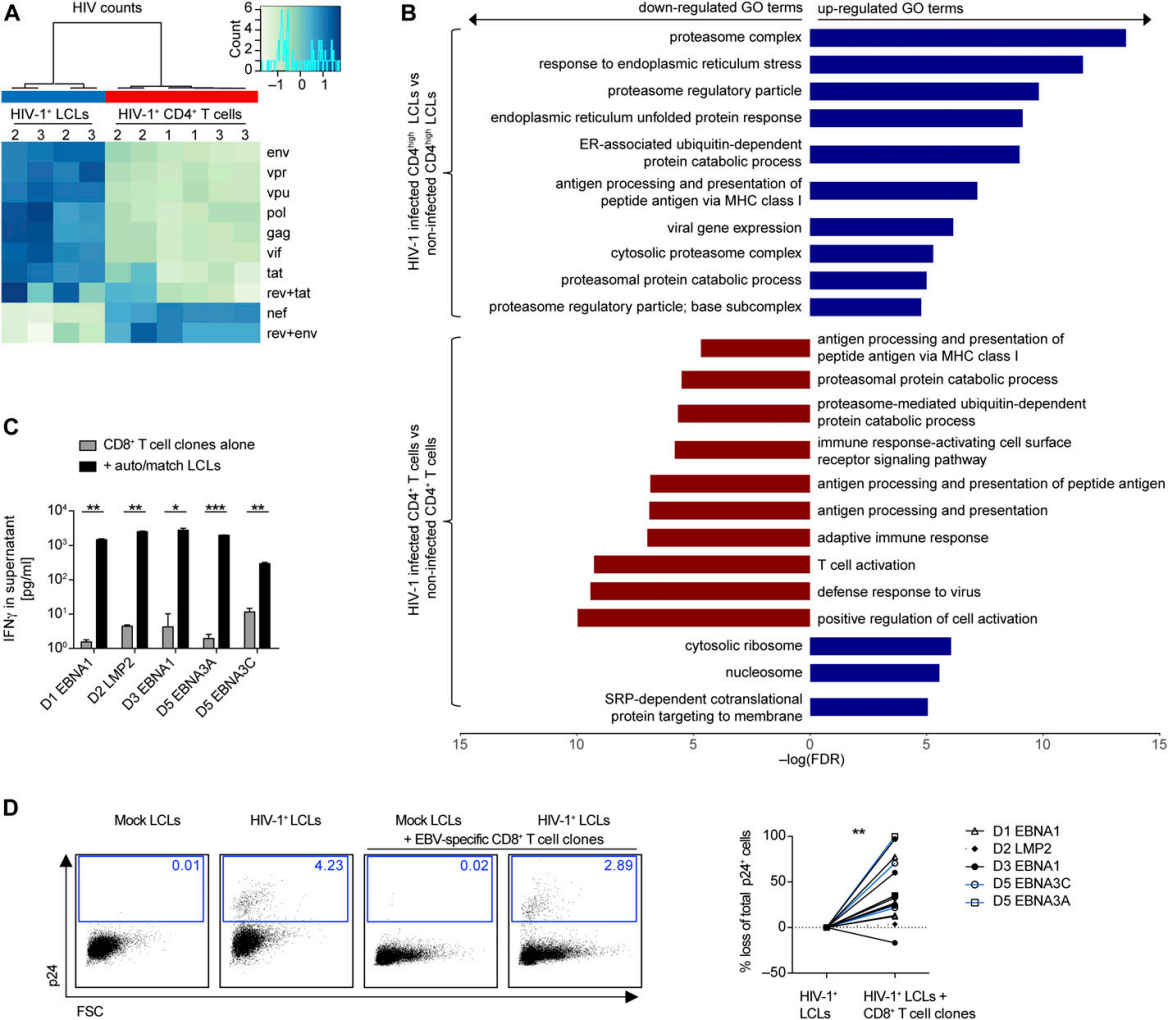
Susceptibility of HIV-1–infected lymphoblastoid cell lines (LCLs) to T-cell clones in vitro. **(A)** Heat map depicting the HIV transcriptome in HIV-1–infected LCLs (2 donors) and three PB CD4^+^ T cells. Two of the CD4^+^ T cells investigated were autologous to the LCLs. Two separate infections were performed for each cell type. **(B)** Bar chart depicting selected results from gene set enrichment analysis of HIV-1–infected T cells and LCLs relative to mock-infected controls. **(A, B)** RNA was extracted 2 d after HIV-1 infection. **(C)** Reactivity of 5 EBV-specific CD8^+^ T-cell clones from four donors with or without autologous or HLA-matched LCLs (auto/match) measured by IFNγ ELISA of the culture supernatant. Donor and specificity for EBV protein: D1 EBNA1, D2 LMP2, D3 EBNA1, D5 EBNA3A, and D5 EBNA3C. Mean ± SD (two-tailed unpaired *t* tests, corrected by the Holm–Sidak method). **(D)** Representative flow cytometry plots from intracellular staining for p24 of mock-infected LCLs, HIV-1–infected LCLs, mock-infected LCLs mixed with an autologous EBV-specific CD8^+^ T-cell clone and HIV-1–infected LCLs mixed with an autologous EBV-specific CD8^+^ T-cell clone indicating percentage of p24-positive of total LCL population. Plots were pre-gated on live, single, total LCLs-labelled before co-culture with a lipophilic membrane dye (PKH67). Specific elimination of p24^+^ autologous or HLA-matched LCLs by individual EBV-specific CD8^+^ T-cell clones expressed as percentage loss of % p24^+^ cells in cocultures with T-cell clones compared with conditions without. Pooled from eight individual experiments, each condition was performed with 2–4 technical replicates (Wilcoxon matched pairs test). **(C, D)** *P*-values are reported as ns *P* > 0.05, *P < 0.05, **P < 0.01, ***P < 0.001.

**Figure S4.**
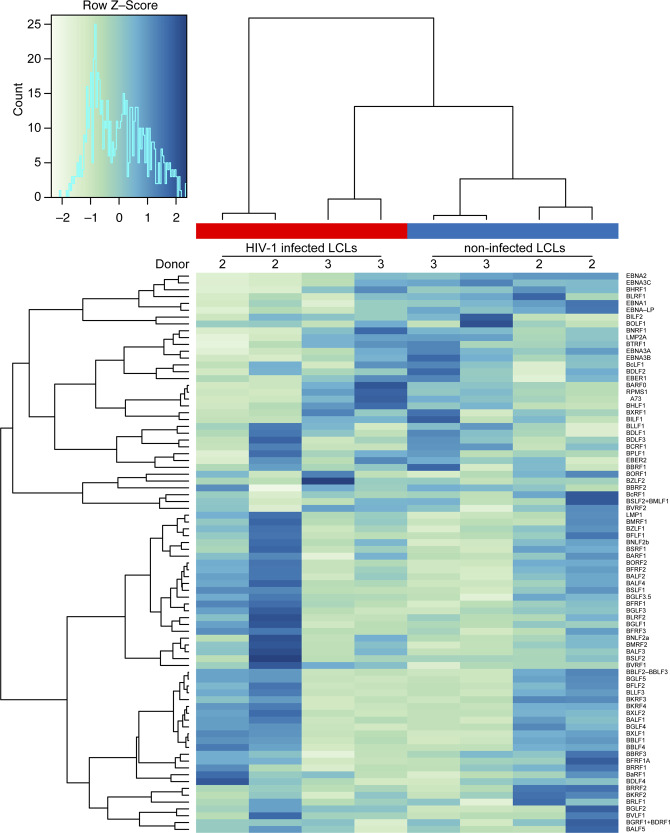
Effect of HIV-1 infection on EBV transcriptome. Heat map of the EBV transcriptome comparing X4-tropic HIV-1-infected lymphoblastoid cell lines 2 d postinfection and noninfected lymphoblastoid cell lines.

**Figure S5.**
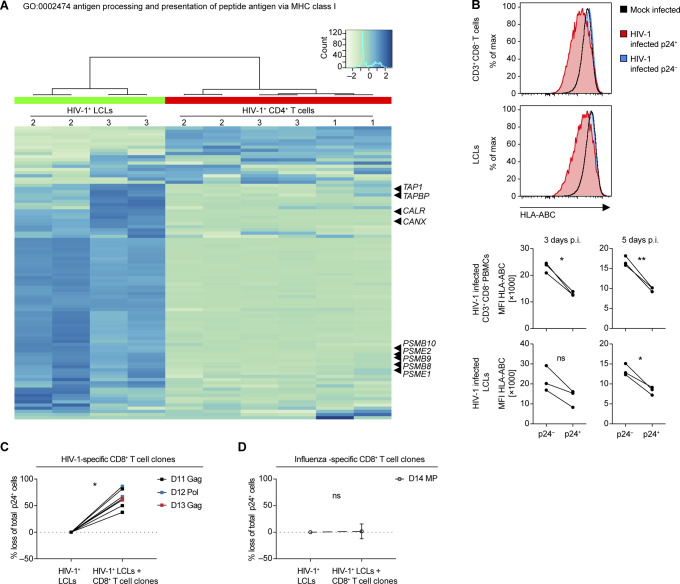
Effect of HIV-1 infection in lymphoblastoid cell lines (LCLs) and T cells on genes in the GO set “0002474 antigen processing and presentation of peptide antigen via MHC class I,” HLA class I surface expression and specific elimination of HIV-1 infected LCLs via HIV-1 specific CD8^+^ T cell clones. **(A)** Heat map of relative gene expression in the gene set “GO:0002474 antigen processing and presentation of peptide antigen via MHC class I” of host transcriptome of HIV-infected LCLs and CD4^+^ T cells. Indicated genes: TAP1 (peptide transporter TAP1), TAPBP (Tapasin), CALR (calreticulin), CANX (calnexin), PSMB10 (Mecl-1), PSME2 (Pa28β), PSMB9 (LMP2), PSMB8 (LMP7), and PSME1 (Pa28α). **(B)** Representative flow cytometry plots 3 d post HIV-1 infection (top panel) and summary of median fluorescence intensity of HLA class I (pan-A/B/C) detected on the surface of mock-infected or HIV-1–infected (p24^−^ and p24^+^ populations) CD3^+^ CD8^−^ T cells and LCLs 3 and 5 d postinfection for three donors (bottom panel) (two-tailed unpaired *t* test). **(C)** Specific elimination of p24^+^ autologous or HLA-matched LCLs by three individual HIV-1–specific CD8^+^ T-cell clones expressed as percentage loss of total p24^+^ cells. **(D)** Specific elimination of p24^+^ LCLs by influenza-specific CD8^+^ T-cell clones (Influenza MP [GIL] - specific) expressed as percentage loss of total p24^+^ cells. Data from two experiments using different (matched) target cells each time. **(C, D)** ns *P* > 0.05, *P < 0.05, **P < 0.01 (Wilcoxon matched-pairs test).

To investigate the effect of HIV infection on the host transcriptome in LCLs and CD4^+^ T cells, the aforementioned RNA sequencing was mined for differentially expressed host and viral genes. This gene expression analysis revealed differential transcription of HIV genes in the two cell types. The nef gene was highly expressed in CD4^+^ T cells compared with other HIV genes and in contrast to the HIV gene expression pattern found in LCLs (Fig 5A). Conversely, hierarchical clustering analysis based on the EBV transcriptome did not show clustering of HIV-1–infected and noninfected LCLs, respectively (Fig S4). In general, HIV-1 affected the host transcriptome of LCLs less than that of CD4^+^ T cells. However, gene set enrichment analysis detected significant disparate expression of genes included in GO sets related to antigen processing and/or presentation of peptide antigen via MHC class I between HIV-1–infected T cells and LCLs relative to mock-infected controls (Fig 5B and Table S5). Specifically, genes related to “antigen processing and presentation of peptide antigen via MHC class I” were significantly down-regulated in HIV-1–infected CD4^+^ T cells and up-regulated in HIV-1–infected LCLs upon GO enrichment analysis. This GO set is composed of 90 genes including genes encoding for TAP1, calreticulin, and subunits of the immunoproteasome, including Mecl-1, the large multifunctional peptidases 2 and 7 (LMP2 and 7), and the proteasome activator components Pa28α and β (Fig S5A). Based on these changes in genes involved in enhanced antigen processing and presentation via MHC class I in LCLs, we investigated whether HIV-1 infection renders LCLs more susceptible to elimination by CD8^+^ T cells. Five EBV-specific CD8^+^ T-cell clones isolated from four EBV^+^ donors with specific reactivity toward endogenously processed EBV latent antigens (Fig 5C) were cultured with autologous or HLA class I–matched X4-tropic HIV-1–infected LCLs. After 18 h of co-culture with the EBV-specific CD8^+^ T-cell clones, the fraction of p24^+^ LCLs in the total LCL population decreased, indicating a more efficient elimination of HIV-1–infected cells than the non–HIV-1–infected LCLs (Fig 5D). Interestingly, cell surface expression levels of HLA class I were down-regulated in both HIV-1–infected LCLs and autologous HIV-1–infected CD4^+^ T cells despite lower relative abundance of nef in LCLs, which has been described to down-regulate MHC class I surface expression (48) (Fig S5B). These data indicate that HIV-1–infected LCLs are highly sensitive to CD8^+^ T-cell immune control despite global HLA class I down-regulation. HIV-1–infected LCLs were also clearly susceptible to HIV-specific CD8^+^ T-cell clones derived from three individual donors (Fig S5C), whereas influenza-specific CD8^+^ T cells did not react to HIV^+^ LCLs preferentially (Fig S5D). Thus, EBV/HIV coinfected B cells appear to be more efficiently targeted by CD8^+^ T cells specific for HIV-1 and EBV in comparison to EBV single-infected B cells. The enhanced vulnerability of EBV-infected B cells upon HIV-1 coinfection may explain why these coinfected cells were preferentially observed upon CD8 depletion in vivo, and point toward HIV-1 superinfected EBV-transformed B cells being efficiently controlled by CD8^+^ T cells, even if their activity to protect from EBV-induced lymphomas is compromised by loss of CD4^+^ T-cell help due to HIV-1 infection.

Table S5 GO term and KEGG analysis of differentially expressed genes in HIV-1–infected and noninfected lymphoblastoid cell lines versus CD4^+^ T cells.

## Discussion

There is increasing evidence that cell types other than CD4^+^ memory T cells have the potential to constitute a fraction of the overall HIV-1 latency reservoir (8, 10). To date, efforts to identify and phenotype cells that make up the HIV-1 reservoir have located HIV-1 provirus in tissue-resident macrophages during cART treatment of a humanized mouse model, in which only human myeloid and B cells develop from a human CD34^+^ HPC graft (49). Furthermore, HPCs have been reported to sustain HIV-1 infection both in vitro and in vivo in patients and in a humanized mouse model (50, 51). We believe that activated B cells could serve as HIV-1 target cells, especially upon infection with the ubiquitous γ-herpesvirus EBV. As such, HIV-1 could potentially directly contribute to malignant transformation or reside latently in the long-lived EBV-transformed memory B cells hidden from the immune system and thus constitute a potential additional long-lived reservoir for HIV-1 latency. Indeed, we found a similar integration profile of X4-tropic HIV-1 in autologous LCLs and CD4^+^ T cells in vitro. Furthermore, we provide evidence that EBV-transformed cells may harbour HIV-1 in vivo after ART treatment, during which no peripheral HIV-1 RNA load can be detected. Dual-infection, however, is likely a rare event as it pertains solely to X4-tropic viruses which occur in only 50% of patients at late-stage disease (52) and only a fraction of the EBV-infected B cells express the requisite surface receptors CD4 and CXCR4. Furthermore, we found that dual-infected cells are highly susceptible to immune control via CD8^+^ T cells in vitro and may only contribute to HIV-1 viremia when CD8^+^ T-cell function is severely impaired. Because we found that the conditions for X4-tropic HIV-1 susceptibility were met in patients with acute EBV infection or EBV-related lymphomas, future studies could investigate whether HIV infection of EBV^+^ B cells occurs in people living with HIV, especially during cART.

We found no evidence that HIV-1 infection perturbs EBV gene expression directly in B cells derived from EBV/HIV dual-infected animals. Similarly, upon in vitro infection of LCLs with HIV-1, the abundance of different EBV transcripts did not indicate a shift in the latency program or an induction of the EBV lytic cycle. This is in contrast to the enhanced EBV lytic gene expression and plasma cell differentiation observed upon EBV/KSHV dual-infection (53). We did observe differential HIV-1 gene expression upon HIV-1 infection in LCLs compared with autologous HIV-1–infected CD4^+^ T cells, which may be explained by different HIV-1 replication dynamics in LCLs compared with T cells (54, 55, 56). This was accompanied by discrete changes in the host transcriptome, whereby genes related to MHC-I–mediated antigen presentation were enriched in HIV-1–infected compared with noninfected LCLs in contrast to HIV-1–infected versus noninfected T cells. Thus, dual-infections of EBV-infected B cells with either HIV-1 or KSHV have a different impact on both EBV and host gene transcription.

Infection of humanized mice with a dose of EBV as used in this study leads to detectable EBV DNA levels, relatively asymptomatic EBV persistence with the development of few EBV-driven tumors and an EBV-specific T-cell response. CD8^+^ T cells expand and infiltrate EBV-associated tumors to a similar degree upon EBV single- and EBV/HIV dual-infection. However, the additional burden of HIV-1 infection resulted in less CD4^+^ T-cell infiltration, an increase in EBV-associated tumors and a trend towards an exhausted CD8^+^ T-cell phenotype. The latter observation has also been made in humanized mouse infections with HIV-1 over longer experimental periods (57). Furthermore, we observed a reduced EBV-specific T-cell response and CD8 depletion did not lead to a higher EBV-associated tumor burden in dual-infected animals. These results, modelled within a very short time frame, mirror the compromised EBV-specific immune control preceding EBV-associated lymphomagenesis that has been observed in HIV-1–infected patient cohorts (6, 7). As such, we find this animal system informative for investigating these human lymphotropic viruses because it recapitulates features of human EBV-specific CD8^+^ T-cell dysfunction associated with HIV-1.

However, some CD8^+^ T-cell function was retained upon dual infection. When CD8^+^ T cells were depleted in EBV/HIV dual-infected mice, an increased number of EBNA2/p24 double-positive cells could be found in the spleen compared with non-depleted dual-infected mice. This points to a preferential immune control of HIV-1 coinfected over EBV single-infected B cells by CD8^+^ T cells. Indeed, latently EBV-infected B cells minimize their recognition by CD8^+^ T cells with low latent EBV antigen expression, resulting in less than one peptide presented on MHC class I molecules per cell for some T-cell epitopes of EBNA3C, the antigen which our HLA-B*4402-restricted CD8^+^ T-cell clone recognized in this study (58). This often allows for only 30% maximal specific lysis by CD8^+^ T-cell clones, such as the HLA-B*0850-restricted EBNA3A-specific T-cell clone used in this study (59). Finally, EBNA1, recognized by two HLA-B*3501-restricted CD8^+^ T-cell clones in this study, limits its own translation and proteasomal degradation through β-sheet formation with its glycine–alanine repeat domain (60, 61). Upon super-infection with replication competent HIV-1, many HIV-1 proteins are expressed, and it is conceivable that a professional antigen-presenting B cell may protect itself from this high protein production by inducing the immunoproteasome by default. As such, the observed transcriptional up-regulation of antigen processing for MHC class I presentation seems to enhance latent EBV antigen recognition by CD8^+^ T cells. The resulting immunoproteasome induction has indeed been described to increase the process of cytosolic proteolysis for optimal MHC class I ligand generation (62, 63, 64, 65, 66). This suggests that HIV-1 coinfection increases antigen processing of viral antigens, rendering the coinfected B cells susceptible to immune control in part because of improved EBV-specific recognition and additionally via HIV-1–specific CD8^+^ T-cell recognition. Thus, HIV-1 can use EBV-infected B cells for integration, replication, and transfer in vivo, but in turn, this target cell for HIV-1 is efficiently controlled by CD8^+^ T cells.

Humanized mice have the ability to recapitulate certain aspects of the human immune system in vivo. However, a number of limitations in immune function should be mentioned at this point, which may preclude direct transfer of these results to human infections (reviewed in reference 25). Importantly, reconstituted human immune system components show similarities to cord blood immune cells. Although cell-mediated immune responses can be mounted, the magnitude of these responses may be lower than in human adults, and isotype switched antibody responses are only rarely observed and steady state levels of IgG are a 1,000-fold lower than in adult human serum. As such, HIV-1 binding on the surface of B cells as immune complexes, as previously described for noninfected B cells (67), may only be poorly modelled in this system. Because EBV-infected cells down-regulate CD21, the extent to which EBV-infected B cells bind immune-complexed HIV-1 via CD21 and thus facilitate infection of susceptible cells, as described previously for non–EBV-infected B cells will have to be further evaluated. Furthermore, this model system cannot fully recapitulate the spatioanatomic aspects of human viral infection and lymph nodes and mucosal secondary lymphoid tissues are poorly developed. In this study, we focused our investigations on particular recombinant strains of type 1 EBV (B95-8) and X4-tropic HIV-1 (NL4-3), respectively. It will be necessary to validate these findings using other EBV and HIV-1 strains in future studies to reflect the strain diversity of both viruses in infected human individuals. Interestingly, type 2 EBV has been described to infect T cells in vitro, in humanized mice, and in healthy infected children, thus expanding further the cellular repertoire within which direct interaction of these two important human pathogens could occur (68, 69, 70).

## Materials and Methods

### Humanized mouse generation and infection

NOD/LtSz-scid IL2Rγnull-tgA2 (NSG-A2, Stock# 009617) and NOD/LtSz-scid IL2Rγnull (NSG, Stock# 005557) mice were obtained from The Jackson Laboratory, bred, and maintained at the Institute of Experimental Immunology, University of Zurich, under specific pathogen-free conditions, with a maximum of five adult animals per cage. Newborn mice (1–5 d old) were irradiated with 1 Gy. 5–7 h after irradiation, mice were injected with 1–3 × 10^5^ CD34^+^ human HPCs derived from HFL tissue obtained from Advanced Bioscience Resources. Isolation of human CD34^+^ cells from HFL tissue was performed as described previously (27, 71) by positive selection for CD34 with magnetic separation according to the manufacturer’s recommendations (Miltenyi Biotec). Both male (n = 48) and female (n = 80) mice were used. Individual mouse cohorts were reconstituted with cells derived from six different donors and NSG-A2 mice were reconstituted specifically with CD34^+^ cells from HLA-A2^+^ HFL samples. Reconstitution of human immune system components in mice was analyzed 3 mo after HPC injection and again 1 wk before the start of the experiments by flow cytometric immune phenotyping of PBMCs for huCD45, huCD3, huCD19, huCD4, huCD8, and HLA-DR as previously described (72). Apart from weighing and tail vein bleeding for analysis of human immune cell reconstitution in peripheral blood, humanized mice were not involved in any procedures before viral infection. Once human immune cell reconstitution was confirmed, humanized mice were injected with 10^4^ Raji Infecting Units (RIUs) of EBV or PBS, and 1 wk later with 0.25 × 10^6^ tissue culture infecting dose 50 (TCID_50_) of HIV-1 or PBS intraperitoneally. For CD8^+^ T-cell depletion, mice received intraperitoneal injections of purified anti-CD8 antibody, clone OKT-8.SUP obtained from hybridoma culture in-house (73) diluted in PBS (total of 150 μg per mouse) at week 2 (1 wk after HIV infection). Experiments were performed with a cohort of mice reconstituted with CD34^+^ cells from a single donor, and animals were distributed to experimental groups to ensure a similar ratio of females to males (62.5% females to 37.5% males) and overall similar human immune reconstitution in the peripheral blood. Animals were euthanized 4 wk post-EBV infection. Investigators were not blinded regarding the viruses used for infection of the animals. Virus-inoculated mice with no EBV DNA load in the blood and spleen and without nuclear EBNA2 staining in formalin-fixed, paraffin-embedded (FFPE) splenic sections or with no HIV RNA load in the peripheral blood and HIV cytoplasmic p24 staining in FFPE splenic sections at 4 wk postinfection were considered noninfected. The tumor frequency in humanized mice was assessed by a tumor score: no macroscopic tumors observed = 0; tumor observed = 1; multiple tumors observed = 2. For autologous cell transfer into humanized mice, see the following paragraphs.

### HIV and EBV virus production

HIV viral stocks were produced by polyethylenimine-mediated transfection (Polysciences) of 293T cells with X4-tropic NL4-3 and HBX2, R5-tropic YU-2 and JR-CSF, and dual-tropic 89.6 plasmid DNA, respectively, provided through the NIH AIDS Research and Reference Reagent Program. 48 h after transfection, virus was harvested, filtered (0.45 μm, TPP syringe filter) and frozen at −80°C until use. Virus titers were determined as previously described (74). Briefly, TCID_50_ was determined by infecting human CD8^+^ T-cell–depleted PBMCs from three donors, which were stimulated by PHA and anti-CD3 beads (Dynal 11131D; Life Technologies). p24 in the postinfection culture supernatants were determined via ELISA. EBV B95-8-GFP (EBVwt, p2089 (75)) was produced in 293 HEK cells and titers of virus concentrates (in RIU) were determined by flow cytometric analysis of GFP^+^ Raji cells 2 d after infection in vitro on a BD FACSCanto II (BD Biosciences) as previously described (72, 76).

### Primary cell cultures

PBMCs were obtained from whole blood of donors after red blood cell removal by density-gradient centrifugation using Ficoll-Paque (GE Healthcare) following the manufacturer’s instructions. CD19^+^ B cells were isolated from PBMCs by magnetic selection using the MACS CD19^+^ isolation kit according to the manufacturer’s instructions (Miltenyi Biotec) after prior incubation with the FcR blocking reagent (included in the CD34^+^ Isolation Kit; Miltenyi Biotec) for 15 min at 4°C. Two MACS columns were used in succession. For the HIV-1 integration site and RNA transcriptome analysis, donor CD4^+^ T cells were isolated by negative magnetic selection using the EasySep human CD4^+^ T-cell isolation kit (Cat. no. 17952; Stemcell Technologies) following the manufacturer’s instructions. LCLs and CD19-depleted PBMCs were cultured in RPMI 1640 (Gibco) medium supplemented with 10% FCS (PAA Laboratories), 50 U/ml penicillin–streptomycin, 25 mM Hepes, 2 mM L-glutamine (R10), and in the case of PBMCs and T cells, 20 IU/ml recombinant IL-2 (Peprotech) was included. CD19^+^ B cells were cultured as previously described (77) in RPMI 1640 (Gibco) medium supplemented with 10% FCS (PAA Laboratories), 50 U/ml penicillin–streptomycin, 25 mM Hepes, 2 mM L-glutamine, IL-4 (10 ng/ml), IL-2 (50 ng/ml), IL-10 (10 ng/ml), and sCD40L (300 ng/ml). All cells were cultured in a humidified incubator at 37°C and 5% CO_2_.

### HIV infection of cells in vitro

For in vitro HIV infections of CD19-depleted PBMCs, purified CD4^+^ T cells were stimulated overnight in R10 supplemented with 5 μg/ml PHA and 20 IU recombinant human IL-2 (Peprotech). Cells were resuspended in HIV virus stocks pre-diluted in R10 at a multiplicity of infection of 1. Cells were centrifuged with the virus at 1,200*g* for 2 h at 24°C, and then washed in PBS. CD19-depleted PBMCs and purified CD4^+^ T cells were maintained in R10 supplemented with 20 IU/ml of recombinant IL-2 (Peprotech). LCLs were maintained in R10. CD19^+^ B cells were maintained in R10 supplemented with IL-4 (10 ng/ml), IL-2 (50 ng/ml), IL-10 (10 ng/ml), and sCD40L (300 ng/ml) as described previously (77). HIV p24 antigen levels in cell culture supernatants were determined using a twin-site sandwich enzyme-linked immunosorbent assay (ELISA) performed essentially as described previously (78). Briefly, a polyclonal antibody was adsorbed to a solid phase to capture p24 antigen from detergent lysates of culture supernatants. Bound p24 was visualized with an alkaline phosphatase–conjugated anti-p24 monoclonal antibody and luminescent detection system. Time point zero for the p24 assay was collected after the final washing step. For in vitro ART experiments, the medium was supplemented with 1 μM Efavirenz and 5 μM AZT (provided through the NIH AIDS Research and Reference Reagent Program) from day 5 postinfection until the end of the experiment on day 10.

### CCR5 genotyping of LCLs

Genotyping was performed as previously described by reference 79. Briefly, PCRs were performed using 1 μl of donor LCL derived DNA and primers flanking the 32-bp deletion: 5′-GTCTTCATTACACCTGCAGCTCTC-3′ and 5′-GTCCAACCTGTTAGAGCTACTGC-3′ (79). DNA was extracted from a known CCR5Δ32 heterozygous individual and included as a control. The amplified products (wild-type CCR5: 311 bp and CCR5Δ32: 279 bp) were analyzed on a 3.0% agarose/TAE gel by electrophoresis and visualized with gel red DNA staining.

### HIV-1 integration site analysis

Genomic DNA and total RNA were extracted from HIV-1 NL4-3–infected and noninfected CD4^+^ T cells and CD4^high^ LCLs (60% CD4^+^) with DNeasy Blood & Tissue kit and RNeasy Mini kit (QIAGEN), respectively, according to the manufacturer’s protocol 2 d post-HIV-1 infection. 5′ HIV-1 integration junctions were amplified from 400 to 1,300 ng of genomic DNA with the nrLAM-PCR (Table S6) (36, 37). A total of 12 pM cell type–specific pooled amplicons were sequenced with the Illumina MiSeq platform using the MiSeq Reagent Kit v2 (300 cycles) (Illumina) with 8% PhiX. Sequencing reads were mapped to the human genome assembly GRCh37.p13 using the Integration Site Analysis Pipeline (InStAP) (37). True 5′ HIV-1 integration junctions were identified based on the following criteria: (i) presence of nrLAM-PCR adaptor and 5′ HIV-1 LTR in the sequencing reads, (ii) host DNA begins within three nucleotides from the end of 5′ HIV-1 LTR, and (iii) ≥98% identity from ≥85% of the sequencing read lengths. Testing for integration of HIV into the EBV DNA genome was performed by utilizing the same bioinformatics pipeline InStAP, exchanging the target genome from the human genome, to the EBV genome (National Center of Biotechnology Information [NCBI] taxid:10376). The default settings for BLAST did not return any hits. The use of BLASTn (suitable for similar sequences) resulted in some hits with similarities that were, however, not statistically significant. Nucleotide conservation plots were prepared using WebLogo (80, 81). Overrepresentation of GO terms among HIV-1 integration sites in genes in NL4-3–infected LCLs and CD4^+^ T cells was performed with a PANTHER Overrepresentation Test (Version 20171205) with the GO database version (2018-02-02) with Fisher’s exact test with FDR multiple test correction.

Table S6 Oligonucleotides.

### RNA transcriptome analysis

RNA was extracted from cells as described in “HIV-1 integration site analysis.” High-throughput sequencing and RNA-seq analysis was performed essentially as previously described (49). Sequenced reads were mapped to a merged genome consisting of EBV (NC_007605.1), HIV-1 (AF324493.2), and Homo Sapiens (GRCh38.p7) using STAR (v2.5.2a) (82). The option “--quantMode GeneCounts” was enabled to count the reads per gene simultaneously, resting upon the annotations from GenBank (EBV and HIV-1) and Ensembl Release 86 (Homo Sapiens). Based on these counts, statistical analysis of differential gene expression was performed with R/Bioconductor package DESeq2 (v1.16.1) (83) because low HIV-1 infection LCL-sample 3–11 was excluded. The obtained *P*-values were corrected for multiple testing by the Benjamini–Hochberg procedure, genes with a mean absolute fold change of minimal 1.5 and a corrected *P*-value lower than 0.1 were considered differentially expressed. For gene set enrichment analysis, R/Bioconductor package gage (v2.20.1) was applied (84), gene sets were obtained from the Gene Ontology Consortium. Gene sets with a Benjamini–Hochberg–corrected *P*-value lower than 0.001 were considered enriched. Heat maps were generated with the normalized counts provided by DESeq2 using R package gplots (v3.0.1). Non–HIV-1–dependent differences between the donors have been removed with R/Bioconductor Package sva (v3.22.0) before plotting, using HIV-1 infection as a surrogate variable. The complete RNA-seq data from this article have been deposited in the European Nucleotide Archive (http://www.ebi.ac.uk/ena). The accession number for the RNA-seq data is PRJEB25772.

### Quantification EBV and HIV burden in humanized mice

EBV infection in mice was monitored by qPCR of DNA from splenic tissue and whole blood. DNA was extracted using DNeasy Blood and Tissue Kit (QIAGEN) and NucliSENS (BioMerieux), respectively, according to the manufacturer’s instructions. Quantitative analysis of EBV DNA in humanized mouse spleens and blood was performed by a TaqMan (Applied Biosystems) real-time PCR as described previously (85) with modified primers (5′-CTTCTCAGTCCAGCGCGTTT-3′ and 5′-CAGTGGTCCCCCTCCCTAGA-3′) and the fluorogenic probe (5′-(FAM)-CGTAAGCCAGACAGCCAATTGTCAG-(TAMRA)-3′) for the amplification of a 70-base pair sequence in the conserved BamHI W fragment of EBV. The PCR was run on an ABI 7300 Thermocycler (Applied Biosystems) and samples analyzed in duplicates. HIV infection in humanized mice was assessed by RT-qPCR for HIV copy numbers in plasma or serum (AmpliPrep/COBAS TaqMan HIV-1 test; Roche). HIV-1 copy numbers in plasma of non-reconstituted NSG mice transplanted with HIV-infected LCLs and humanized controls were assessed by an in-house RT-qPCR assay. 60 μl of plasma or serum of mice was diluted in 80 μl of water. RNA was extracted following the QIAamp Viral RNA Mini Kit manufacturer’s instructions (QIAGEN). Reverse transcription was performed using 12 μl of RNA using the SKCC1B primer 5′-TACTAGTAGTTCCTGCTATGTCACTTCC-3′ (86). The reaction was performed in a final volume of 20 μl:0.25 μM SKCC1B primer, 4 μl 5× iScript selection reaction mix, 2 μl gene specific primer enhancer solution, 1 μl iScript reverse transcriptase (Bio-Rad). The thermal cycler was set to 42°C for 60 min followed by inactivation at 85°C for 5 min. The real-time quantitative PCR was performed using 3 μl of cDNA prepared during the reverse transcription step as previously described (87) using the ts5′gag 5′-CAAGCAGCCATGCAAATGTTAAAAGA-3′ and SKCC1B primers as well as the mf319tq 5′-TGCAGCTTCCTCATTGATGGT-3′ probe. The real-time quantitative PCR reaction was performed in a final volume of 12.5 μl:3.8 μM of each primer, 1.15 μM of the probe, and 6.25 μl DreamTaq Hot Start PCR master mix. Thermal cycling was performed with a C1000 Touch CFX384 Real-Time platform (Bio-Rad) starting with 95°C for 4 min and 50 cycles of the following steps: denaturation at 95°C for 5 s, annealing at 55°C for 5 s, and elongation at 60°C for 30 s.

### Serum cytokine quantification

Serum aliquots were collected during euthanizing animals and stored at −80°C until use. Cytokine concentrations in the serum were determined with the V-plex Mesoscale Discovery Human Proinflammatory Panel 1 (Cat. no. K15049D) and was performed as per the manufacturer’s instructions in duplicates; standard dilutions of the calibrator blend were performed in quadruplicates.

### Flow cytometry

Total white blood cells in whole blood were counted using a Beckman cell counter according to the manufacturer’s instructions. Mouse whole blood was collected in K2 EDTA tubes (BD) and lysed twice with ACK lysis buffer (Lonza) for 5 min and washed in PBS. Mouse splenocyte cell suspensions were obtained by mechanical disruption of the spleen through a 70-μm nylon mesh, lysis with ACK lysis buffer for 10 min, and washing with PBS. Cells from mouse whole blood and spleens or in vitro–cultured cells were labelled with mixtures of fluorochrome-labelled antihuman monoclonal antibodies in PBS (Table S7). Acquisitions were performed on an LSR Fortessa (BD) flow cytometer and data were analyzed with FlowJo (v9). Cellular debris and dead cells were excluded by their light-scattering characteristics, as well as fixable live dead stains.

Table S7 Monoclonal Antibodies.

### RT-qPCR

Total RNA was isolated from in vitro–infected B cell lines or humanized mice derived CD19^+^ splenocytes using the RNeasy Mini Kit (QIAGEN) according to the manufacturer’s instructions. Contaminating genomic DNA was removed with a 15-min on-column DNase treatment during RNA isolation (RNase-Free DNase Set; QIAGEN). The purified RNA was immediately reverse-transcribed with GoScript Reverse Transcriptase (Promega) according to the manufacturer’s recommendations in a 20-μl volume for 1 h at 42°C using a primer mix combining previously described 3′ gene-specific RT primers (53, 88) and reverse primers for HIV transcripts listed in Table S6 at concentrations of 10 μM each. After 15-min heat inactivation at 70°C, 0.2 U RNase H (Thermo Fisher Scientific) was added for 20 min at 37°C, followed by RNase H heat inactivation for 20 min at 65°C and freezing of the cDNA at −20°C. Amplifications of *Cp/Wp-EBNA1*, *EBNA2*, *LMP1*, *LMP2A*, *BZLF1*, *GAPDH*, and *SDHA* were carried out in triplicate with equal volumes of input RNA and TaqMan universal PCR reagents (Applied Biosystems) using either previously published primer sets with 5′FAM/3′TAMRA (88) or 5′FAM/3′MGB labelled probes for *SDHA* (TaqMan Applied Biosystems Gene Expression Assay [Hs00417200]). Primers and probes for quantification of HIV-unspliced, unspliced and single-spliced, and multiple-spliced transcripts (89, 90) are listed in Table S6. Thermal cycling was performed with a C1000 Touch CFX384 Real-Time platform (Bio-Rad) starting with 2 min at 50°C and 10 min at 95°C, followed by 50 cycles of amplification (95°C for 15 s and 60°C for 1 min). Cq values were determined with the CFX-manager software (Bio-Rad) using a regression algorithm. Levels of EBV transcripts in B cells from infected humanized mice were calculated relative to the geometric mean of the two reference genes, *GAPDH* and *SDHA*, and then normalized to the mean value of the EBV^+^ single-infected animals. Mock-infected control animals were negative for EBV transcripts. For HIV-1 splice variant RNA quantification in infected LCLs, absolute HIV-1 transcript numbers were calculated using dilutions of an all-in-one HIV-1 standard DNA containing the HIV-1 RNA splice variants as described and calculated previously (91).

### EBV-specific IFN-γ release assay (ELISPOT)

EBV-specific T-cell responses were analyzed using an IFNγ ELISpot assay, essentially as previously described (27, 92). Briefly, splenocytes were depleted of human CD19^+^ cells using anti-CD19 microbeads (Miltenyi Biotec). The CD19-depleted fraction was stimulated with autologous LCLs at a ratio of 1:4 for 18 h and incubation with R10 and PMA/ionomycin served as negative and positive controls, respectively. Each condition was performed in duplicates or triplicates. Spots were counted on an ELISPOT reader system (ELR02; Autoimmun Diagnostika GmbH).

### Immunohistochemistry

Immunohistochemistry (IHC) was performed on sections obtained from FFPE-humanized mouse spleen and tumor tissue with a BOND-MAX automated IHC system (Leica Microsystems). Sections of 3 μm from FFPE blocks were deparaffinized, and then antigen retrieval was performed by incubation in Bond Epitope Retrieval Solution 2 (ER2; Leica) for 30 min at 100°C followed by incubation with antibodies against p24 (M0857, dil. 1:40; Dako), EBNA2 (Ab90543, dil. 1:200; Abcam Ltd.), CD20 (120R-16, dil.1:300; Cell Marque Lifescreen Ltd.), CD4 (104R-16, dil.1:100; Cell Marque Lifescreen Ltd.), or CD8 (108R-16, 1:500; Cell Marque Lifescreen Ltd.), respectively, which were detected with the Refine-DAB-Kit (Leica). For double IHC of p24 and EBNA2, DAB^+^ p24–stained sections were incubated in ER2 for 10 min at 95°C before incubation with the mouse anti-EBNA2 antibody, which was detected with the Refine AP-Kit and new fast red (Leica). For double IHC of PAX5 and p24, sections were incubated in ER2 for 20 min at 95°C before incubation with the mouse anti-PAX5 antibody (PA0552, dil. 1:20; Leica) for 30 min, which was detected with the Bond Polymer Refine Detection Kit and DAB (Leica), then incubated with the mouse anti-p24 antibody (M0857, dil. 1:10; Dako) for 30 min, which was detected with the Bond Polymer Refine Red Detection Kit and new fast red (Leica). Nuclei were counterstained with hematoxylin (AP refine Kit; Leica or JT Baker Hämatoxylin; Leica). EBER in situ hybridization and CD4 co-immunohistochemistry was performed on 3 μm FFPE PTLD sections from a tissue microarray block (34) using a Benchmark Ultra automated slide stainer (Ventana). Briefly, tissue sections were pretreated with protease 3 before incubation with an EBER-specific probe (Ventana), which was detected via the ISH iView Blue Detection Kit (Ventana). Subsequently, tissue sections were incubated with cell conditioner 1 for antigen retrieval, rabbit monoclonal antibody against human CD4 (clone SP35), and visualization performed with OptiView DAB IHC Detection Kit (Ventana). All images presented in this article were white set point adjusted with Adobe Ps CS5 V12.1.

### Quantification of immunohistochemistry

PAX5/p24 and EBNA2/p24 co-stainings were analyzed with a Vectra3 automated quantitative pathology imaging system (PerkinElmer) using Vectra and InForm software (PerkinElmer). Images were acquired via an automated scanning protocol created with InForm tissue segmentation to recognize the tissue. Images were acquired with 20× objective lens with a CCD camera using the scanning protocol. Images taken were used to set up algorithms in inForm to recognize and count PAX5/p24– or EBNA2/p24–positive cells, respectively. For comparison of tumor tissue and non-tumorous spleen, the regions of interest were defined manually. Single chromogen control stains were used to eliminate signal cross-talk. The number of positive cells was determined per 1 mm^2^.

### Autologous transfer experiments

Once human immune cell reconstitution was confirmed, 3 mo after reconstitution, donor humanized mice were injected i.p. with 10^5^ EBV RIU or PBS and 1 wk later with 5 × 10^5^ NL4-3 HIV-1 TCID_50_ or PBS. Mice received 150 μg OKT8 antibody (purification described below) or PBS i.p. at week 2 (1 wk after HIV infection) and at week 4. Animals were euthanized between 5 and 6 wk post-EBV infection. Spleens of donor mice were mashed through a 0.75 μM nylon mesh followed by red blood cell lysis using ACK buffer (Lonza) as per the manufacturer’s instructions. CD19^+^ B cells were isolated from total splenocytes as described for PBMCs above. The labelled B-cell fraction was passed over two MACS columns in succession. The non-labelled T-cell–containing CD19-depleted fraction was also collected. Purity was confirmed via flow cytometry immunophenotyping. Equivalent numbers of CD19^+^ B cells or CD19-depleted splenocytes were injected into autologously reconstituted (same HFL donor-derived CD34^+^ HPCs) recipient humanized mice (in one case the respective cell fractions from two mice were pooled). Autologous HPC-reconstituted recipient animals were chosen to avoid an allogenic T-cell response. Recipient mice were euthanized 6–7 wk post-donor cell transfer.

### HIV-1 latency experiments

Non-reconstituted 9–18-wk-old NSG mice received 1–2 × 10^5^ in vitro NL4-3 HIV-1–infected LCLs via i.p. injection. LCLs were derived from EBV-infected humanized mice via ex vivo outgrowth in R10 medium. The B cell identity of these LCLs was confirmed via RNA-seq (Sample E4-3 (53)). Control humanized mice or non-reconstituted NSG mice were injected i.p. with 5 × 10^5^ NL4-3 HIV-1 TCID_50_. cART was applied via the drinking water in MediDrop Sucralose solution, and briefly abacavir (ABC) (100 mg/kg/day), lamivudine (3TC) (150 mg/kg/day), and dolutegravir (DTG) (15 mg/kg/day) pills were reduced to powder and solubilized in MediDrop Sucralose solution for 15 min on a magnetic stirrer. Final amount of drugs per 500 ml of MediDrop Sucralose solution was 0.54 g of ABC, 0.622 g of 3TC, and 1.86 g of DTG. Control humanized mice received cART 4 wk post-HIV-1 infection for 2 wk and then were switched back to normal drinking water for 2 wk. LCL-bearing mice received cART on the day of HIV-1^+^ LCL transfer for 2 wk and then were switched back to normal drinking water for 2 wk. All animals were euthanized 4 wk post-start of cART treatment. Spleens and tumor material of mice were disrupted mechanically through a 0.75-μM nylon mesh followed by red blood cell lysis of splenocytes using ACK buffer (Lonza) as per the manufacturer’s instructions. Total genomic DNA (for ALU-gag LTR qPCR) and RNA (for HIV-1 copy number RT-qPCR) was simultaneously extracted using the RNeasy Midi Kit (QIAGEN) as previously described (93). Briefly, the disrupted spleen and tumor material was resuspended in RLT lysis buffer (QIAGEN) as per the manufacturer’s instructions. After RNA isolation, genomic DNA was eluted from the RNeasy gel-silica membrane by addition of DNA- and nuclease-free isolation buffer (8.0 mM NaOH). Columns were left at 55°C for 10 min and centrifuged at 5,000*g* for 3 min at room temperature. pH of samples was adjusted with Hepes to pH 7–8 and supplemented with 1.0 mM EDTA.

### HIV-1 Alu-PCR integration assay

Genomic DNA were extracted from splenocytes of HIV-1 NL4-3–infected humanized NSG mice and from tumors derived from NSG mice engrafted with HIV-1 NL4-3–infected LCLs. A two-step PCR amplification was performed as described (94). The first PCR amplification was performed on 300 ng of genomic DNA, or no template controls with the genomic *Alu* forward 5′-GCC TCC CAA AGT GCT GGG ATT ACA G-3′ and HIV gag reverse 5′-GTT CCT GCT ATG TCA CTT CC-3′ primers. The reactions were performed in 50 μl: 1× PCR buffer, 1.5 mM MgCl_2_, 0.3 mM dNTPs, 0.1 μM *Alu* forward primer, 0.6 μM gag reverse primer, and 0.05 units of Jump start Taq DNA polymerase/μl (Sigma-Aldrich). The thermal cycler ABI 7500 (Applied Biosystems) was programmed to perform a 2-min hot start at 95°C and then 20 cycles of the following steps: denaturation at 95°C for 30 s, annealing at 50°C for 30 s, and elongation at 72°C for 3 min and 30 s with a final extension at 75°C for 5 min. The second-round real-time quantitative PCR was performed using 10 μl of the reaction from the first PCR amplification with the LTR (R) forward 5′-TTA AGC CTC AAT AAA GCT TGC C-3′; LTR (U5) reverse, 5′-GTT CGG GCG CCA CTG CTA GA-3′ primers. The wild-type probe primer sequence was 5′-FAM-CCA GAG TCA CAC AAC AGA GGG GCA CA-TAMRA-3′. Reaction was carried out in 20 μl reaction mix that contained 1× PCR buffer, 3.5 mM MgCl_2_, 0.005 μM carboxy-X-rhodamine (ROX; Molecular Probes), 0.3 mM dNTPs, 0.25 μM LTR forward and reverse primers, 0.1 μM wild-type probe, and 0.125 units of JumpStart Taq DNA polymerase/μl (Sigma-Aldrich). The reactions were performed on an ABI 7500 instrument (Applied Biosystems) with the following thermal program: 5 min hot start at 95°C and then 50 cycles of the following steps: denaturation at 95°C for 15 s and annealing and elongation at 60°C for 30 s. The ABI 7500 measurement point was at elongation 60°C-30 s.

### Purification of mouse antihuman CD8 antibody

The OKT-8 hybridoma cells (mouse IgG2a; ATCC) (73) were gradually adapted to the serum- and protein-free PFHM-II medium (Cat. no. 12040-077; Thermo Fisher Scientific, [formerly Gibco]). Adapted cells were cultured in BD CELLineTM 1000 flasks (Cat. no. 353137; BD) according to the manufacturer’s recommendation. Antibodies were precipitated from the culture supernatant by addition of an equal volume of saturated ammonium-sulfate solution. After buffer exchange to PBS using PD-10 desalting columns (Sephadex G25 medium; GE Healthcare), antibodies were sterilized by filtration through 0.2 μm (Filtropur S 0.2; Sarstedt) and stored at 4°C until use. Antibody purity was assessed by PAGE and Coomassie Brilliant Blue staining, protein concentration was determined by OD280 nm measurement (using an extinction coefficient of 1.37 (at 1 g/l) for concentration calculation) and CD8-binding was confirmed by flow cytometry.

### CD8^+^ T-cell cloning and restimulation

The LMP2_426–434_–specific CD8^+^ T-cell clone that detects the CLGGLLTMV epitope presented on HLA-A*0201 used in this study has previously been described (72). CD8^+^ T-cell clones specific for EBNA1 were generated from freshly isolated PBMCs which were depleted of CD4^+^ T cells using anti-CD4 conjugated MACS microbeads and magnetic selection (Miltenyi Biotec). Additional CD8^+^ T-cell clones specific for EBNA3A and –C were generated without prior CD4^+^ T-cell depletion. PBMCs were stimulated with 5 μM of EBNA1 HPVGEADYFEY peptide or EBNA3A–C peptide mix (EBNA3A: FLRGRAYGL, RPPIFIRRL, YPLHEQHGM, RLRAEAQVK; EBNA3B: IVTDFSVIK; and EBNA3C: RRIYDLIEL, EENLLDFVRF) for three to 4 h. Responding cells were enriched by IFN-γ secretion assay (130-054-201; Miltenyi Biotec) and were cloned by limiting dilution at 3 and 30 cells per well on HLA-matched gamma-irradiated LCLs (10^4^/well) loaded with the relevant peptide or peptide mix at 5 μM and allogeneic gamma-irradiated, PHA-treated PBMCs (10^5^/well) in IL-2-supplemented (150 IU/ml) medium with 10% pooled human sera (T cell medium). Growing microcultures were screened for peptide reactivity by IFN-γ ELISA (3420-1H-20; Mabtech). Selected CD8^+^ T-cell clones were expanded on autologous gamma-irradiated LCLs loaded with the relevant peptide at 5 μM and allogeneic gamma-irradiated, PHA-treated PBMCs in T-cell medium. Clones were maintained in the T-cell medium. Every 2–3 wk, CD8^+^ T-cell clones were restimulated with gamma-irradiated peptide-pulsed autologous LCLs and allogenic PHA-treated PBMCs. To control for specific recognition of target cells, 5,000 CD8^+^ T-cell clones were co-cultured with 50,000 autologous LCLs. After overnight incubation, co-culture supernatants from single wells were tested by ELISA for IFN-γ content. The resulting additional CD8^+^ T cell clones used in this study were specific for EBNA1_407-417_ (HPVGEADYFEY, HLA-B*3501, clones from two donors), EBNA3A_325-333_ (FLRGRAYGL, HLA-B*0850), and EBNA3C_281-290_ (EENLLDFVRF, HLA-B*4402). The CD8^+^ T-clone–specific for Influenza MP_(58-66)_ GILGFVFTL presented on HLA-A*0201 described previously (95) was kindly provided by Jean-Francois Fonteneau, Nantes, France. The CD8^+^ T clones D11 and D12 specific for HIV-1 gag peptide SLYNTVATL and HIV-1 pol peptide ILKEPVHGV presented on HLA-A*0201, respectively, were kindly provided by Yanchun Peng and Tao Dong, Oxford, UK. The CD8^+^ T clone D13 specific for HIV-1 gag peptide SLYNTVATL presented on HLA-A*0201 was kindly provided by Arnaud Moris, Paris, France.

### In vitro killing assay

Cytotoxic activity of CD8^+^ EBV-specific T-cell clones against autologous or HLA class I–matched HIV-1–infected LCLs was assessed by in vitro killing assays. X4-tropic HIV-1–infected LCLs and noninfected LCLs were labelled with a lipophilic membrane dye (PKH67, Cat. no. MINI67; Sigma-Aldrich, or CellVue Claret Far Red, Cat. no. MINCLARET-1KT; Sigma-Aldrich) according to the manufacturer’s instructions. Infected and noninfected LCLs were incubated with EBNA1-, EBNA3A-, EBNA3C-, or LMP2-specific CD8^+^ T-cell clones for 18 h at an effector to the target ratio of 5:1. Cells were stained with the Zombie NIR live cell/dead cell discrimination dye (Cat. no. 423105; BioLegend) and treated with the BD fixation/permeabilization kit for intracellular staining of HIV-1 p24 core antigen using the HIV-1 p24 (Beckman Coulter) antibody. Specific lysis of HIV^+^ LCLs was determined by using the formula: % lysis = 100 ×([% p24^+^ of PKH67^+^ LCLs without effectors − % p24^+^ of PKH67^+^ LCLs with effectors]/% p24^+^ PKH67^+^ LCLs without effectors). All culture conditions were run at least in duplicates and when possible in triplicates, or higher replicates.

### Statistics

Data were analyzed using GraphPad Prism software (v8.3.1). Normally distributed data were analyzed with a two-tailed *t* test, with Welch’s correction if variances were significantly different as determined by the f test. Paired data sets were compared with a two-tailed paired *t* test or Wilcoxon matched pairs test. Mann–Whitney test was used for non-normally distributed data to compare the means of data sets. Multiple *t* tests were corrected by the Holm–Sidak method. Two-tailed chi-squared (n > 10,000) test or Fisher’s exact test (n < 10,000) was used for categorical data. Differences were considered significant at *P* < 0.05 in two-tailed tests. For analysis of RNA sequencing data, see RNA transcriptome analysis.

### Study approval

Studies using HFL samples as well as peripheral blood samples of healthy HIV-negative donors were reviewed and approved by the Cantonal Ethics Committee of Zurich, Switzerland (protocols no. KEK-StV-Nr.19/08 and 2019-00837). Studies using blood samples of EBV^+^ PTLD donors and EBV^+^ infectious mononucleosis or control donors, upon receipt of written informed consent, were reviewed and approved by the Institutional Review Board of the University of Hong Kong/Hospital Authority Hong Kong West Cluster (IRB reference no. UW 16-508 and EC 1940-02). The study of PTLD patient tissue microarray material was reviewed and approved by the Ethics Committee of Northwestern and Central Switzerland (protocol no. EKNZ 2014-252). Research was conducted in accordance with the Declaration of Helsinki. Animal protocols were approved by the veterinary office of the canton of Zurich, Switzerland (protocol nos. 148/2011, 209/2014, 93/2014, and 81/2017).

## Supplementary Material

Reviewer comments

## References

[1] Cesarman E (2011) Gammaherpesvirus and lymphoproliferative disorders in immunocompromised patients. Cancer Lett 305: 163–174. 10.1016/j.canlet.2011.03.003 21493001 PMC3742547

[2] Luzuriaga K, Sullivan JL (2010) Infectious mononucleosis. New Engl J Med 362: 1993–2000. 10.1056/nejmcp1001116 20505178

[3] Xu GJ, Kula T, Xu Q, Li MZ, Vernon SD, Ndung’u T, Ruxrungtham K, Sanchez J, Brander C, Chung RT, (2015) Viral immunology. Comprehensive serological profiling of human populations using a synthetic human virome. Science 348: aaa0698 10.1126/science.aaa0698 26045439 PMC4844011

[4] Kutok JL, Wang F (2006) Spectrum of Epstein-Barr virus-associated diseases. Annu Rev Pathol 1: 375–404. 10.1146/annurev.pathol.1.110304.100209 18039120

[5] Douek DC, Roederer M, Koup RA (2009) Emerging concepts in the immunopathogenesis of AIDS. Annu Rev Med 60: 471–484. 10.1146/annurev.med.60.041807.123549 18947296 PMC2716400

[6] Gasser O, Bihl FK, Wolbers M, Loggi E, Steffen I, Hirsch HH, Gunthard HF, Walker BD, Brander C, Battegay M, (2007) HIV patients developing primary CNS lymphoma lack EBV-specific CD4^+^ T cell function irrespective of absolute CD4^+^ T cell counts. PLoS Med 4: 556–561. 10.1371/journal.pmed.0040096 PMC1831733 17388662

[7] Piriou E, van Dort K, Nanlohy NM, van Oers MHJ, Miedema F, van Baarle D (2005) Loss of EBNA1-specific memory CD4^+^ and CD8^+^ T cells in HIV-infected patients progressing to AIDS-related non-hodgkin lymphoma. Blood 106: 3166–3174. 10.1182/blood-2005-01-0432 16014568

[8] Kandathil AJ, Sugawara S, Balagopal A (2016) Are T cells the only HIV-1 reservoir? Retrovirology 13: 86 10.1186/s12977-016-0323-4 27998285 PMC5175311

[9] Honeycutt JB, Wahl A, Baker C, Spagnuolo RA, Foster J, Zakharova O, Wietgrefe S, Caro-Vegas C, Madden V, Sharpe G, (2016) Macrophages sustain HIV replication in vivo independently of T cells. J Clin Invest 126: 1353–1366. 10.1172/jci84456 26950420 PMC4811134

[10] Sattentau QJ, Stevenson M (2016) Macrophages and HIV-1: An unhealthy constellation. Cell Host Microbe 19: 304–310. 10.1016/j.chom.2016.02.013 26962941 PMC5453177

[11] Moore JP, Kitchen SG, Pugach P, Zack JA (2004) The CCR5 and CXCR4 coreceptors-central to understanding the transmission and pathogenesis of human immunodeficiency virus type 1 infection. AIDS Res Hum Retroviruses 20: 111–126. 10.1089/088922204322749567 15000703

[12] Gras G, Legendre C, Krzysiek R, Dormont D, Galanaud P, Richard Y (1996) CD40/CD40L interactions and cytokines regulate HIV replication in B cells in vitro. Virology 220: 309–319. 10.1006/viro.1996.0319 8661382

[13] Gras G, Richard Y, Roques P, Olivier R, Dormont D (1993) Complement and virus-specific antibody-dependent infection of normal B lymphocytes by human immunodeficiency virus type 1. Blood 81: 1808–1818. 10.1182/blood.v81.7.1808.1808 8461467

[14] Moir S, Lapointe R, Malaspina A, Ostrowski M, Cole CE, Chun TW, Adelsberger J, Baseler M, Hwu P, Fauci AS (1999) CD40-mediated induction of CD4 and CXCR4 on B lymphocytes correlates with restricted susceptibility to human immunodeficiency virus type 1 infection: Potential role of B lymphocytes as a viral reservoir. J Virol 73: 7972–7980. 10.1128/jvi.73.10.7972-7980.1999 10482544 PMC112811

[15] Poulin L, Paquette N, Moir S, Lapointe R, Darveau A (1994) Productive infection of normal CD40-activated human B lymphocytes by HIV-1. AIDS 8: 1539–1544. 10.1097/00002030-199411000-00004 7531456

[16] Casareale D, Sinangil F, Hedeskog M, Ward W, Volsky DJ, Sonnabend J (1983) Establishment of retrovirus-, Epstein-Barr virus-positive B-lymphoblastoid cell lines derived from individuals at risk for acquired immune deficiency syndrome (AIDS). AIDS Res 1: 253–270. 10.1089/aid.1.1983.1.253 6085940

[17] Fritsch L, Marechal V, Schneider V, Barthet C, Rozenbaum W, Moisan-Coppey M, Coppey J, Nicolas JC (1998) Production of HIV-1 by human B cells infected in vitro: Characterization of an EBV genome-negative B cell line chronically synthetizing a low level of HIV-1 after infection. Virology 244: 542–551. 10.1006/viro.1998.9120 9601522

[18] Gras GS, Dormont D (1991) Antibody-dependent and antibody-independent complement-mediated enhancement of human immunodeficiency virus type 1 infection in a human, Epstein-Barr virus-transformed B-lymphocytic cell line. J Virol 65: 541–545. 10.1128/jvi.65.1.541-545.1991 1845908 PMC240554

[19] Laurence J, Astrin SM (1991) Human immunodeficiency virus induction of malignant transformation in human B lymphocytes. Proc Natl Acad Sci U S A 88: 7635–7639. 10.1073/pnas.88.17.7635 1652756 PMC52356

[20] Rodriguez-Alfageme C, Chen Z, Sonoda G, Testa JR, Damiani RD, Astrin SM (1998) B cells malignantly transformed by human immunodeficiency virus are polyclonal. Virology 252: 34–38. 10.1006/viro.1998.9445 9875314

[21] Tozzi V, Britton S, Ehrnst A, Lenkei R, Strannegard O (1989) Persistent productive HIV infection in EBV-transformed B lymphocytes. J Med Virol 27: 19–24. 10.1002/jmv.1890270105 2537875

[22] Montagnier L, Gruest J, Chamaret S, Dauguet C, Axler C, Guetard D, Nugeyre MT, Barre-Sinoussi F, Chermann JC, Brunet JB, (1984) Adaptation of lymphadenopathy associated virus (LAV) to replication in EBV-transformed B lymphoblastoid cell lines. Science 225: 63–66. 10.1126/science.6328661 6328661

[23] Schnittman SM, Lane HC, Higgins SE, Folks T, Fauci AS (1986) Direct polyclonal activation of human B lymphocytes by the acquired immune deficiency syndrome virus. Science 233: 1084–1086. 10.1126/science.3016902 3016902

[24] Moir S, Fauci AS (2009) B cells in HIV infection and disease. Nat Rev Immunol 9: 235–245. 10.1038/nri2524 19319142 PMC2779527

[25] Lysenko V, McHugh D, Behrmann L, Rochat MA, Wilk CM, Kovtonyuk L, Bourquin JP, Münz C, Manz MG, Speck R, (2017) Humanised mouse models for haematopoiesis and infectious diseases. Swiss Med Wkly 147: w14516 10.4414/smw.2017.14516 29120027

[26] Yajima M, Imadome K, Nakagawa A, Watanabe S, Terashima K, Nakamura H, Ito M, Shimizu N, Yamamoto N, Fujiwara S (2009) T cell-mediated control of Epstein-Barr virus infection in humanized mice. J Infect Dis 200: 1611–1615. 10.1086/644644 19832115

[27] Strowig T, Gurer C, Ploss A, Liu YF, Arrey F, Sashihara J, Koo G, Rice CM, Young JW, Chadburn A, (2009) Priming of protective T cell responses against virus-induced tumors in mice with human immune system components. J Exp Med 206: 1423–1434. 10.1084/jem.20081720 19487422 PMC2715061

[28] Caduff N, McHugh D, Murer A, Ramer P, Raykova A, Landtwing V, Rieble L, Keller CW, Prummer M, Hoffmann L, (2020) Immunosuppressive FK506 treatment leads to more frequent EBV-associated lymphoproliferative disease in humanized mice. PLoS Pathog 16: e1008477 10.1371/journal.ppat.1008477 32251475 PMC7162544

[29] De Rossi A, Roncella S, Calabro ML, D’Andrea E, Pasti M, Panozzo M, Mammano F, Ferrarini M, Chieco-Bianchi L (1990) Infection of Epstein-Barr virus-transformed lymphoblastoid B cells by the human immunodeficiency virus: Evidence for a persistent and productive infection leading to B cell phenotypic changes. Eur J Immunol 20: 2041–2049. 10.1002/eji.1830200924 2170147

[30] Monroe JE, Calender A, Mulder C (1988) Epstein-Barr virus-positive and -negative B-cell lines can be infected with human immunodeficiency virus types 1 and 2. J Virol 62: 3497–3500. 10.1128/jvi.62.9.3497-3500.1988 2841499 PMC253476

[31] Nikitin PA, Yan CM, Forte E, Bocedi A, Tourigny JP, White RE, Allday MJ, Patel A, Dave SS, Kim W, (2010) An ATM/Chk2-mediated DNA damage-responsive signaling pathway suppresses Epstein-Barr virus transformation of primary human B cells. Cell Host Microbe 8: 510–522. 10.1016/j.chom.2010.11.004 21147465 PMC3049316

[32] Hoennscheidt C, Max D, Richter N, Staege MS (2009) Expression of CD4 on Epstein-Barr virus-immortalized B cells. Scand J Immunol 70: 216–225. 10.1111/j.1365-3083.2009.02286.x 19703011

[33] Mrozek-Gorska P, Buschle A, Pich D, Schwarzmayr T, Fechtner R, Scialdone A, Hammerschmidt W (2019) Epstein-Barr virus reprograms human B lymphocytes immediately in the prelatent phase of infection. Proc Natl Acad Sci U S A 116: 16046–16055. 10.1073/pnas.1901314116 31341086 PMC6690029

[34] Menter T, Dickenmann M, Juskevicius D, Steiger J, Dirnhofer S, Tzankov A (2017) Comprehensive phenotypic characterization of PTLD reveals potential reliance on EBV or NF-kappaB signalling instead of B-cell receptor signalling. Hematol Oncol 35: 187–197. 10.1002/hon.2280 26799990

[35] Freed EO (2001) HIV-1 replication. Somat Cell Mol Genet 26: 13–33. 10.1023/a:1021070512287 12465460

[36] Paruzynski A, Arens A, Gabriel R, Bartholomae CC, Scholz S, Wang W, Wolf S, Glimm H, Schmidt M, von Kalle C (2010) Genome-wide high-throughput integrome analyses by nrLAM-PCR and next-generation sequencing. Nat Protoc 5: 1379–1395. 10.1038/nprot.2010.87 20671722

[37] Kok YL, Vongrad V, Shilaih M, Di Giallonardo F, Kuster H, Kouyos R, Gunthard HF, Metzner KJ (2016) Monocyte-derived macrophages exhibit distinct and more restricted HIV-1 integration site repertoire than CD4^+^ T cells. Sci Rep 6: 24157 10.1038/srep24157 27067385 PMC4828718

[38] Lewinski MK, Bisgrove D, Shinn P, Chen H, Hoffmann C, Hannenhalli S, Verdin E, Berry CC, Ecker JR, Bushman FD (2005) Genome-wide analysis of chromosomal features repressing human immunodeficiency virus transcription. J Virol 79: 6610–6619. 10.1128/jvi.79.11.6610-6619.2005 15890899 PMC1112149

[39] Schroder AR, Shinn P, Chen H, Berry C, Ecker JR, Bushman F (2002) HIV-1 integration in the human genome favors active genes and local hotspots. Cell 110: 521–529. 10.1016/s0092-8674(02)00864-4 12202041

[40] Barr SD, Ciuffi A, Leipzig J, Shinn P, Ecker JR, Bushman FD (2006) HIV integration site selection: Targeting in macrophages and the effects of different routes of viral entry. Mol Ther 14: 218–225. 10.1016/j.ymthe.2006.03.012 16647883

[41] Demeulemeester J, Vets S, Schrijvers R, Madlala P, De Maeyer M, De Rijck J, Ndung’u T, Debyser Z, Gijsbers R (2014) HIV-1 integrase variants retarget viral integration and are associated with disease progression in a chronic infection cohort. Cell Host Microbe 16: 651–662. 10.1016/j.chom.2014.09.016 25525795

[42] Stevens SW, Griffith JD (1996) Sequence analysis of the human DNA flanking sites of human immunodeficiency virus type 1 integration. J Virol 70: 6459–6462. 10.1128/jvi.70.9.6459-6462.1996 8709282 PMC190680

[43] Wang GP, Ciuffi A, Leipzig J, Berry CC, Bushman FD (2007) HIV integration site selection: Analysis by massively parallel pyrosequencing reveals association with epigenetic modifications. Genome Res 17: 1186–1194. 10.1101/gr.6286907 17545577 PMC1933515

[44] Marini B, Kertesz-Farkas A, Ali H, Lucic B, Lisek K, Manganaro L, Pongor S, Luzzati R, Recchia A, Mavilio F, (2015) Nuclear architecture dictates HIV-1 integration site selection. Nature 521: 227–231. 10.1038/nature14226 25731161

[45] Feederle R, Neuhierl B, Bannert H, Geletneky K, Shannon-Lowe C, Delecluse HJ (2007) Epstein-Barr virus B95.8 produced in 293 cells shows marked tropism for differentiated primary epithelial cells and reveals interindividual variation in susceptibility to viral infection. Int J Cancer 121: 588–594. 10.1002/ijc.22727 17417777

[46] van Baarle D, Hovenkamp E, Callan MF, Wolthers KC, Kostense S, Tan LC, Niesters HG, Osterhaus AD, McMichael AJ, van Oers MH, (2001) Dysfunctional Epstein-Barr virus (EBV)-specific CD8^+^ T lymphocytes and increased EBV load in HIV-1 infected individuals progressing to AIDS-related non-Hodgkin lymphoma. Blood 98: 146–155. 10.1182/blood.v98.1.146 11418474

[47] Gorantla S, Makarov E, Finke-Dwyer J, Gebhart CL, Domm W, Dewhurst S, Gendelman HE, Poluektova LY (2010) CD8(+) cell depletion accelerates HIV-1 immunopathology in humanized mice. J Immunol 184: 7082–7091. 10.4049/jimmunol.1000438 20495069 PMC3008566

[48] Schwartz O, Marechal V, Le Gall S, Lemonnier F, Heard JM (1996) Endocytosis of major histocompatibility complex class I molecules is induced by the HIV-1 Nef protein. Nat Med 2: 338–342. 10.1038/nm0396-338 8612235

[49] Honeycutt JB, Thayer WO, Baker CE, Ribeiro RM, Lada SM, Cao Y, Cleary RA, Hudgens MG, Richman DD, Garcia JV (2017) HIV persistence in tissue macrophages of humanized myeloid-only mice during antiretroviral therapy. Nat Med 23: 638–643. 10.1038/nm.4319 28414330 PMC5419854

[50] Sebastian NT, Zaikos TD, Terry V, Taschuk F, McNamara LA, Onafuwa-Nuga A, Yucha R, Signer RAJ, Riddell Iv J, Bixby D, (2017) CD4 is expressed on a heterogeneous subset of hematopoietic progenitors, which persistently harbor CXCR4 and CCR5-tropic HIV proviral genomes in vivo. PLoS Pathog 13: e1006509 10.1371/journal.ppat.1006509 28732051 PMC5540617

[51] Nixon CC, Vatakis DN, Reichelderfer SN, Dixit D, Kim SG, Uittenbogaart CH, Zack JA (2013) HIV-1 infection of hematopoietic progenitor cells in vivo in humanized mice. Blood 122: 2195–2204. 10.1182/blood-2013-04-496950 23886835 PMC3785119

[52] Connor RI, Sheridan KE, Ceradini D, Choe S, Landau NR (1997) Change in coreceptor use correlates with disease progression in HIV-1-infected individuals. J Exp Med 185: 621–628. 10.1084/jem.185.4.621 9034141 PMC2196142

[53] McHugh D, Caduff N, Barros MHM, Rämer P, Raykova A, Murer A, Landtwing V, Quast I, Styles CT, Spohn M, (2017) Persistent KSHV infection increases EBV-associated tumor formation in vivo via enhanced EBV lytic gene expression. Cell Host Microbe 22: 61–73. 10.1016/j.chom.2017.06.009 28704654

[54] Collin M, Gordon S (1994) The kinetics of human immunodeficiency virus reverse transcription are slower in primary human macrophages than in a lymphoid cell line. Virology 200: 114–120. 10.1006/viro.1994.1169 7510432

[55] Fernandez-Larsson R, Srivastava KK, Lu S, Robinson HL (1992) Replication of patient isolates of human immunodeficiency virus type 1 in T cells: A spectrum of rates and efficiencies of entry. Proc Natl Acad Sci U S A 89: 2223–2226. 10.1073/pnas.89.6.2223 1549586 PMC48629

[56] Srivastava KK, Fernandez-Larsson R, Zinkus DM, Robinson HL (1991) Human immunodeficiency virus type 1 NL4-3 replication in four T-cell lines: Rate and efficiency of entry, a major determinant of permissiveness. J Virol 65: 3900–3902. 10.1128/jvi.65.7.3900-3902.1991 1674969 PMC241424

[57] Palmer BE, Neff CP, LeCureux J, Ehler A, DSouza M, Remling-Mulder L, Korman AJ, Fontenot AP, Akkina R (2013) In vivo blockade of the PD-1 receptor suppresses HIV-1 viral loads and improves CD4^+^ T cell levels in humanized mice. J Immunol 190: 211–219. 10.4049/jimmunol.1201108 23209326 PMC3529847

[58] Crotzer VL, Christian RE, Brooks JM, Shabanowitz J, Settlage RE, Marto JA, White FM, Rickinson AB, Hunt DF, Engelhard VH (2000) Immunodominance among EBV-derived epitopes restricted by HLA-B27 does not correlate with epitope abundance in EBV-transformed B-lymphoblastoid cell lines. J Immunol 164: 6120–6129. 10.4049/jimmunol.164.12.6120 10843661

[59] Subklewe M, Sebelin K, Block A, Meier A, Roukens A, Paludan C, Fonteneau JF, Steinman RM, Münz C (2005) Dendritic cells expand Epstein Barr virus specific CD8^+^ T cell responses more efficiently than EBV transformed B cells. Hum Immunol 66: 938–949. 10.1016/j.humimm.2005.07.003 16360833

[60] Levitskaya J, Coram M, Levitsky V, Imreh S, Steigerwald-Mullen PM, Klein G, Kurilla MG, Masucci MG (1995) Inhibition of antigen processing by the internal repeat region of the Epstein-Barr virus nuclear antigen-1. Nature 375: 685–688. 10.1038/375685a0 7540727

[61] Yin Y, Manoury B, Fahraeus R (2003) Self-inhibition of synthesis and antigen presentation by Epstein-Barr virus-encoded EBNA1. Science 301: 1371–1374. 10.1126/science.1088902 12958359

[62] Basler M, Kirk CJ, Groettrup M (2013) The immunoproteasome in antigen processing and other immunological functions. Curr Opin Immunol 25: 74–80. 10.1016/j.coi.2012.11.004 23219269

[63] Dick TP, Ruppert T, Groettrup M, Kloetzel PM, Kuehn L, Koszinowski UH, Stevanovic S, Schild H, Rammensee HG (1996) Coordinated dual cleavages induced by the proteasome regulator PA28 lead to dominant MHC ligands. Cell 86: 253–262. 10.1016/s0092-8674(00)80097-5 8706130

[64] Gaczynska M, Rock KL, Goldberg AL (1993) Gamma-interferon and expression of MHC genes regulate peptide hydrolysis by proteasomes. Nature 365: 264–267. 10.1038/365264a0 8396732

[65] Groettrup M, Soza A, Eggers M, Kuehn L, Dick TP, Schild H, Rammensee HG, Koszinowski UH, Kloetzel PM (1996) A role for the proteasome regulator PA28alpha in antigen presentation. Nature 381: 166–168. 10.1038/381166a0 8610016

[66] Kincaid EZ, Che JW, York I, Escobar H, Reyes-Vargas E, Delgado JC, Welsh RM, Karow ML, Murphy AJ, Valenzuela DM, (2011) Mice completely lacking immunoproteasomes show major changes in antigen presentation. Nat Immunol 13: 129–135. 10.1038/ni.2203 22197977 PMC3262888

[67] Moir S, Malaspina A, Li Y, Chun TW, Lowe T, Adelsberger J, Baseler M, Ehler LA, Liu S, Davey RT Jr, (2000) B cells of HIV-1-infected patients bind virions through CD21-complement interactions and transmit infectious virus to activated T cells. J Exp Med 192: 637–646. 10.1084/jem.192.5.637 10974030 PMC2193277

[68] Coleman CB, Wohlford EM, Smith NA, King CA, Ritchie JA, Baresel PC, Kimura H, Rochford R (2015) Epstein-Barr virus type 2 latently infects T cells, inducing an atypical activation characterized by expression of lymphotactic cytokines. J Virol 89: 2301–2312. 10.1128/jvi.03001-14 25505080 PMC4338898

[69] Coleman CB, Daud II, Ogolla SO, Ritchie JA, Smith NA, Sumba PO, Dent AE, Rochford R (2017) Epstein-Barr virus type 2 infects T cells in healthy Kenyan children. J Infect Dis 216: 670–677. 10.1093/infdis/jix363 28934430 PMC5853903

[70] Coleman CB, Lang J, Sweet LA, Smith NA, Freed BM, Pan Z, Haverkos B, Pelanda R, Rochford R (2018) Epstein-Barr virus type 2 infects T cells and induces B cell lymphomagenesis in humanized mice. J Virol 92: e00813–e00818. 10.1128/jvi.00813-18 30089703 PMC6189503

[71] White RE, Ramer PC, Naresh KN, Meixlsperger S, Pinaud L, Rooney C, Savoldo B, Coutinho R, Bodor C, Gribben J, (2012) EBNA3B-deficient EBV promotes B cell lymphomagenesis in humanized mice and is found in human tumors. J Clin Invest 122: 1487–1502. 10.1172/jci58092 22406538 PMC3314448

[72] Antsiferova O, Müller A, Rämer P, Chijioke O, Chatterjee B, Raykova A, Planas R, Sospedra M, Shumilov A, Tsai MH, (2014) Adoptive transfer of EBV specific CD8^+^ T cell clones can transiently control EBV infection in humanized mice. PLoS Pathog 10: e1004333 10.1371/journal.ppat.1004333 25165855 PMC4148450

[73] Reinherz EL, Kung PC, Goldstein G, Levey RH, Schlossman SF (1980) Discrete stages of human intrathymic differentiation: Analysis of normal thymocytes and leukemic lymphoblasts of T-cell lineage. Proc Natl Acad Sci U S A 77: 1588–1592. 10.1073/pnas.77.3.1588 6966400 PMC348542

[74] McDougal JS, Cort SP, Kennedy MS, Cabridilla CD, Feorino PM, Francis DP, Hicks D, Kalyanaraman VS, Martin LS (1985) Immunoassay for the detection and quantitation of infectious human retrovirus, lymphadenopathy-associated virus (LAV). J Immunol Methods 76: 171–183. 10.1016/0022-1759(85)90489-2 2981931

[75] Delecluse HJ, Hilsendegen T, Pich D, Zeidler R, Hammerschmidt W (1998) Propagation and recovery of intact, infectious Epstein-Barr virus from prokaryotic to human cells. Proc Natl Acad Sci U S A 95: 8245–8250. 10.1073/pnas.95.14.8245 9653172 PMC20961

[76] Chijioke O, Muller A, Feederle R, Barros MH, Krieg C, Emmel V, Marcenaro E, Leung CS, Antsiferova O, Landtwing V, (2013) Human natural killer cells prevent infectious mononucleosis features by targeting lytic Epstein-Barr virus infection. Cell Rep 5: 1489–1498. 10.1016/j.celrep.2013.11.041 24360958 PMC3895765

[77] Bovia F, Salmon P, Matthes T, Kvell K, Nguyen TH, Werner-Favre C, Barnet M, Nagy M, Leuba F, Arrighi JF, (2003) Efficient transduction of primary human B lymphocytes and nondividing myeloma B cells with HIV-1-derived lentiviral vectors. Blood 101: 1727–1733. 10.1182/blood-2001-12-0249 12406892

[78] Moore JP, McKeating JA, Weiss RA, Sattentau QJ (1990) Dissociation of gp120 from HIV-1 virions induced by soluble CD4. Science 250: 1139–1142. 10.1126/science.2251501 2251501

[79] Glass WG, McDermott DH, Lim JK, Lekhong S, Yu SF, Frank WA, Pape J, Cheshier RC, Murphy PM (2006) CCR5 deficiency increases risk of symptomatic West Nile virus infection. J Exp Med 203: 35–40. 10.1084/jem.20051970 16418398 PMC2118086

[80] Crooks GE, Hon G, Chandonia JM, Brenner SE (2004) WebLogo: A sequence logo generator. Genome Res 14: 1188–1190. 10.1101/gr.849004 15173120 PMC419797

[81] Schneider TD, Stephens RM (1990) Sequence logos: A new way to display consensus sequences. Nucleic Acids Res 18: 6097–6100. 10.1093/nar/18.20.6097 2172928 PMC332411

[82] Dobin A, Davis CA, Schlesinger F, Drenkow J, Zaleski C, Jha S, Batut P, Chaisson M, Gingeras TR (2013) STAR: Ultrafast universal RNA-seq aligner. Bioinformatics 29: 15–21. 10.1093/bioinformatics/bts635 23104886 PMC3530905

[83] Love MI, Huber W, Anders S (2014) Moderated estimation of fold change and dispersion for RNA-seq data with DESeq2. Genome Biol 15: 550 10.1186/s13059-014-0550-8 25516281 PMC4302049

[84] Luo W, Friedman MS, Shedden K, Hankenson KD, Woolf PJ (2009) GAGE: Generally applicable gene set enrichment for pathway analysis. BMC Bioinformatics 10: 161 10.1186/1471-2105-10-161 19473525 PMC2696452

[85] Berger C, Day P, Meier G, Zingg W, Bossart W, Nadal D (2001) Dynamics of Epstein-Barr virus DNA levels in serum during EBV-associated disease. J Med Virol 64: 505–512. 11468736

[86] Michael NL, Herman SA, Kwok S, Dreyer K, Wang J, Christopherson C, Spadoro JP, Young KKY, Polonis V, McCutchan FE, (1999) Development of calibrated viral load standards for group M subtypes of human immunodeficiency virus type 1 and performance of an improved AMPLICOR HIV-1 MONITOR test with isolates of diverse subtypes. J Clin Microbiol 37: 2557–2563. 10.1128/jcm.37.8.2557-2563.1999 10405401 PMC85282

[87] Nischang M, Sutmuller R, Gers-Huber G, Audige A, Li D, Rochat MA, Baenziger S, Hofer U, Schlaepfer E, Regenass S, (2012) Humanized mice recapitulate key features of HIV-1 infection: A novel concept using long-acting AntiRetroviral drugs for treating HIV-1. PloS One 7: e38853 10.1371/journal.pone.0038853 22719966 PMC3374767

[88] Bell AI, Groves K, Kelly GL, Croom-Carter D, Hui E, Chan AT, Rickinson AB (2006) Analysis of Epstein-Barr virus latent gene expression in endemic Burkitt’s lymphoma and nasopharyngeal carcinoma tumour cells by using quantitative real-time PCR assays. J Gen Virol 87: 2885–2890. 10.1099/vir.0.81906-0 16963746

[89] Schmid A, Gianella S, von Wyl V, Metzner KJ, Scherrer AU, Niederost B, Althaus CF, Rieder P, Grube C, Joos B, (2010) Profound depletion of HIV-1 transcription in patients initiating antiretroviral therapy during acute infection. PloS One 5: e13310 10.1371/journal.pone.0013310 20967271 PMC2953504

[90] Althaus CF, Gianella S, Rieder P, von Wyl V, Kouyos RD, Niederost B, Schmid A, Metzner KJ, Joos B, Gunthard HF, (2010) Rational design of HIV-1 fluorescent hydrolysis probes considering phylogenetic variation and probe performance. J Virol Methods 165: 151–160. 10.1016/j.jviromet.2010.01.012 20116399

[91] Strouvelle VP, Braun DL, Vongrad V, Scherrer AU, Kok YL, Kouyos RD, Stockle M, Rauch A, Darling K, Hoffmann M, (2018) No effect of pegylated interferon-alpha on total Hiv-1 DNA load in HIV-1/HCV coinfected patients. J Infect Dis 217: 1883–1888. 10.1093/infdis/jiy131 29528417

[92] Bickham K, Goodman K, Paludan C, Nikiforow S, Tsang ML, Steinman RM, Münz C (2003) Dendritic cells initiate immune control of epstein-barr virus transformation of B lymphocytes in vitro. J Exp Med 198: 1653–1663. 10.1084/jem.20030646 14657218 PMC2194129

[93] Martins WK (2009) A useful procedure to isolate simultaneoulsy DNA and RNA from a single tumor. Protoc Exch. 10.1038/nprot.2009.163

[94] Yu JJQ, Wu TL, Liszewski MK, Dai JH, Swiggard WJ, Baytop C, Frank I, Levine BL, Yang W, Theodosopoulos T, (2008) A more precise HIV integration assay designed to detect small differences finds lower levels of integrated DNA in HAART treated patients. Virology 379: 78–86. 10.1016/j.virol.2008.05.030 18649912 PMC2610678

[95] Fonteneau JF, Larsson M, Somersan S, Sanders C, Münz C, Kwok WW, Bhardwaj N, Jotereau F (2001) Generation of high quantities of viral and tumor-specific human CD4^+^ and CD8^+^ T-cell clones using peptide pulsed mature dendritic cells. J Immunol Methods 258: 111–126. 10.1016/s0022-1759(01)00477-x 11684128

